# Phenotyping for lodging tolerance in pearl millet: identification of optimal environment, plant stage, and potential traits

**DOI:** 10.3389/fpls.2026.1879275

**Published:** 2026-09-28

**Authors:** Shashi Kumar Gupta, Ponnaiah Govintharaj, Chinna Ghouse Peera Shaikh Kulsum, Jana Kholová, Kakkadan Ayyathan Jayalekha, Yogendra Singh Verma

**Affiliations:** 1 International Crops Research Institute for the Semi-Arid Tropics (ICRISAT), Patancheru, Telangana, India; 2 Department of Information Technologies, Faculty of Economics and Management, Czech University of Life Sciences, Prague, Czechia; 3 Bayer Bioscience Pvt. Ltd., Secundrabad, India; 4 Metahelix Life Sciences Ltd., Telangana, Medak, India

**Keywords:** digital plant culm strength meter, lodging tolerance, panicle girth, plant culm strength, stem lodging

## Abstract

Crop lodging poses a significant threat to global food security by reducing crop yields in terms of quantity and quality and causing difficulties in the harvesting process. Rainy-season crops like pearl millet encounter high-velocity winds and rain during different stages of crop growth, thus leading to drastic reductions in grain yields due to crop lodging. The aim of this study was (i) to identify the optimal environment for phenotyping, (ii) to identify trait linkages with lodging tolerance, and (iii) to identify the appropriate plant growth stages for lodging phenotyping. Hence, a four-year (2016–2019) field study was conducted at ICRISAT-India, involving 29 released and pipeline pearl millet single-cross hybrids procured from 11 seed companies and evaluated for several shoot and root traits to generate basic information to help phenotyping pearl millet for lodging tolerance. The study identified two traits, plant culm strength (PCS) and panicle girth (PG), which can differentiate between lodging-tolerant and lodging-susceptible cultivars irrespective of weather conditions. The studies indicated that lodging-tolerant and lodging-susceptible entries showed significant differences in PCS and PG values under well-watered (WW) conditions compared with water-stressed (WS) conditions. Investigations at different plant growth stages (anthesis, milky-grain, and grain-maturity) indicated that PCS and PG estimated at milky-grain and grain-maturity stages provide better differentiation between lodging-tolerant and lodging-susceptible cultivars. PCS was found to be positively correlated with culm diameter. Overall, this study provided preliminary results for phenotype pearl millet for lodging stress.

## 1 Introduction

High-yielding cultivars are a necessity, but plants should have the ability to withstand the forces of nature, like high-speed winds and rains, so that grains can finally be harvested in good condition. Lodging tolerance in plants is a much-desired trait to improve the productivity of agriculturally important food crops. Generally, lodging occurs in the form of stem lodging, root lodging, or both in most cereal crops (Berry et al., 2003a; Wu and Ma, 2016; Robertson et al., 2017) and in some other crops like soybean (Cooper, 1981), tomato (Adelana, 1980), and tobacco (Walton and Casada, 1979). Stem lodging is defined as the permanent bending of crop stems leaving one or two internodes from ground level, whereas root lodging is referred to as the failure of roots to maintain strong soil contact. Both forms of lodging result in lower grain yield, reduced grain quality, and difficulty in harvesting (Wu and Ma, 2019).

Lodging is a complex trait that is affected by various factors such as field management practices (fertilizers and planting density), environmental factors (wind, hail, rain, and soil type), morphological traits, biochemical composition, and anatomical structures of the culm. Resistance to stem lodging can be determined by several morphological traits, including reduced plant height (Curtis and Halford, 2014) and stem thickness of basal internodes (Pinthus, 1973; Kashiwagi and Ishimaru, 2004; Xiao et al., 2015). Anatomical traits like stem/culm wall thickness, the number and size of vascular bundles, and the thickness of the sclerenchyma layer (Kong et al., 2013), and biochemical traits such as structural carbohydrates like cellulose, hemicellulose, and lignin contents of stalks have been reported to be closely linked with stem lodging (Pinthus, 1973; Zhang et al., 2016). In addition, root traits have been found to be associated with root lodging resistance in various crops (Crook and Ennos, 1994; Berry et al., 2003a). For example, a wider spread angle has been found to be positively correlated with root lodging tolerance (Pinthus, 1967).

Several methods have been developed and proposed to phenotype stalk/stem lodging resistance in various crop plants in the field and laboratory (Berry et al., 2003b; Flint-Garcia et al., 2003a; Peiffer et al., 2013). Lodging rate, the ratio of the number of lodged plants to the total number of plants per plot area, has been shown to be adversely associated with stalk lodging resistance and is considered the most direct measure to assess stalk lodging resistance (Xue et al., 2017). Plant standability score is another widely used method to assess cultivars for lodging tolerance (Berry et al., 2003a; Kelbert et al., 2004). Recently, unmanned aerial vehicles (UAVs) have been successfully used to screen cultivars for lodging tolerance in large nurseries (Singh et al., 2019). All these screening methods are limited by weather conditions.

It has previously been shown that, in the absence of extreme weather conditions, measuring stalk/stem strength is the most reliable indicator for determining lodging resistance in crop plants. For example, a stalk-crushing strength tester was initially developed and tested for lodging resistance by measuring the force needed to break the stalk/stem using a hydraulic press (Zuber and Grogan, 1961). Another testing methodology for lodging resistance is the rind penetration test (rind puncture resistance), which involves piercing the stalk/stem rind with a small probe that is attached to a digital force gauge (Flint-Garcia et al., 2003a, b; Peiffer et al., 2013) and measuring the maximum force required to penetrate the rind. Stalk flexural stiffness, a three-point bending mechanical test inspired by structural engineering beam theory, is used to characterize the mechanical strength of the stem (Robertson et al., 2016). These methods are convenient to use and efficient in measuring stalk resistance, but all have been standardized under laboratory conditions; therefore, their accuracy may not be the same as under field conditions. To overcome these limitations, several researchers have used field-based testing devices like a plant culm strength meter (hand-held prostrate testing device) in winter wheat (Xiao et al., 2015), canola (Wu and Ma, 2016), and rice (Kashiwagi and Ishimaru, 2004). Recently, researchers have used portable wind tunnels and mobile wind machines (devised by DuPont Pioneer and Beijing Research Center for Information Technology in Agriculture) to evaluate lodging resistance (Sterling et al., 2003; Shrestha et al., 2020). DuPont Pioneer has devised a mobile wind machine that can generate wind speeds up to 45 m s^−1^ to assess stalk lodging under controlled wind conditions and thus facilitate the evaluation of a genotype for lodging in maize (Barreiro et al., 2008; Wen et al., 2019).

Pearl millet is mainly grown during the rainy season in the rainfed areas of India and Africa, where it often encounters stormy winds or incessant rain, or both, making this crop more vulnerable to lodging (**Supplementary Figure 1**). In the past, breeders have mainly focused on breeding cultivars exclusively for high yield and tolerance to biotic and abiotic stresses in pearl millet. In a feedback exercise conducted with plant breeders from about 50 organizations from both the public and private sectors in India, lodging tolerance was identified as one of the top three traits that need attention in future pearl millet breeding programs (Jayalekha et al., 2016). Researchers face challenges in identifying practical and most reliable methods for determining lodging tolerance mechanisms in crop plants due to its dependency on weather conditions. Breeding programs have been categorizing entries as lodging-tolerant or lodging-susceptible based on natural lodging incidence under field conditions. Natural screening is highly unreliable because of its dependence on weather conditions, creating a need to identify non-weather-dependent tools/traits to estimate lodging tolerance.

Therefore, the present study aimed to conduct preliminary investigations of agro-morphological, biochemical, and root traits associated with lodging tolerance in pearl millet and to develop a non-weather-dependent screening methodology to phenotype pearl millet for lodging tolerance under field conditions.

## 2 Materials and methods

### 2.1 Plant materials

A total of 29 released and pipeline pearl millet hybrid entries obtained from 11 seed companies were used in this study to identify lodging tolerance-associated traits across diverse seasons over four consecutive years (2016 to 2019) at the International Crops Research Institute for the Semi-Arid Tropics (ICRISAT), Patancheru (18° N, 78° E, 545 m above sea level) (**Supplementary Table 1**). These hybrids were grouped into two categories, lodging-tolerant (LT) and lodging-susceptible (LS), based on the feedback received from seed companies. All these experiments were conducted on Alfisol soils at ICRISAT, Patancheru, India. The soil characteristics of the ICRISAT experimental farm are presented in **Supplementary Table 2**. The rainfall and temperature data for the site during the experimental period are provided in **Table 1** (Bhattacharyya et al., 2016). 

**Table 1:** Characteristics of weather parameters at the experimental site (ICRISAT, Patancheru).

Experiment	Year	Crop season	Overall rainfall (mm)	Temperature (°C) (Mean values over the crop season)	
				Maximum	Minimum
Experiment I	2016	Rainy (July to October)	842.6	30.3	19.9
Experiment II	2017	Summer (February to May)	17.4	39.7	14.4
Experiment III	2018	Rainy (July to October)	336.2	32.2	18.7
Experiment IV	2019	Summer (February to May)	58.2	41.4	17.6
Experiment V	2019	Rainy (July to October)	679.4	31.9	20.7

### 2.2 Identification of traits affecting lodging

#### 2.2.1 Experiment I: rainy season of 2016

Experiment I was initiated during the rainy season of 2016, with four known lodging-tolerant and three known lodging-susceptible hybrid entries (based on the feedback received from seed companies). They were evaluated for lodging tolerance-linked morpho-physiological traits at the anthesis, milky-grain, and grain-maturity stages. The trial was planted in a completely randomized block design with two replications. The trial plot consisted of four rows, each 4 m long, with rows spaced 75 cm apart. Experimental plots were fertilized with nitrogen and phosphorus fertilizers as a basal dose in the form of 100 kg ha^-1^ of diammonium phosphate (18% N and 46% P) at the time of field preparation. The same plots were fertilized once with a rate of 100 kg ha^-1^ of urea (46% N) as a top dressing when plants were about knee-high. Trials were irrigated at intervals of 12 to 15 days as required; the crop was protected from diseases and pests during the whole cropping period.

At the anthesis, milky-grain, and grain-maturity stages, plant height (PH, cm) was measured from the base of the stem to the tip of the panicle of the main stem; the number of tillers per plant (NT) was counted as the total number of tillers in the plant; culm diameter (CD) was recorded between the middle portion of the second and third internode from the ground using a digital vernier caliper; and the dry weight of different plant parts (stem, leaf, tillers, productive tillers, and panicle) was measured after 15 days of oven drying at 60 °C. The dried samples of the main stem and vegetative/productive tiller were ground in a Thomas Wiley mill (Model No. 4, Philadelphia, USA) through a 1-mm screen for biochemical analysis. The ground main stem and vegetative/productive tiller samples were analyzed for acid detergent lignin (ADL) by near-infrared reflectance spectroscopy (NIRS) as described by Bidinger and Blümmel (2007) and Blümmel et al. (2007). Cellulose and hemicellulose of the main stem and vegetative/productive tiller were determined according to Van Soest and Robertson (1979). Further, productive tillers (PT) were counted at the milky-grain stage, panicle length (PL, cm) was measured from the base to the tip of the main tiller panicle, and panicle girth (PG, mm) was measured at the center of the panicle of the main tiller at the grain-maturity stage. In addition, lodging percentage (%) was calculated at maturity by dividing the total number of lodged plants by the total number of plants in the plot.

To measure the plant culm strength of the entries, we used a digital plant culm/stem strength meter (Model YYD-1B, Zhe Jhiang top cloud-Agri Technology CO., LTD, China; approximately USD 449 in 2026). The “U”-shaped probe (for tearing down the upright crop culm) was attached to the front of the digital plant stem strength meter before measurement and placed perpendicular to the main stem of the plant at a height of 15 cm above the soil surface. The probe was then pushed down to the right to an angle of 45° with respect to the vertical, and the pressure was measured in kilopascals (kPa) (**Figures 1A, B**).

**Figure 1:**
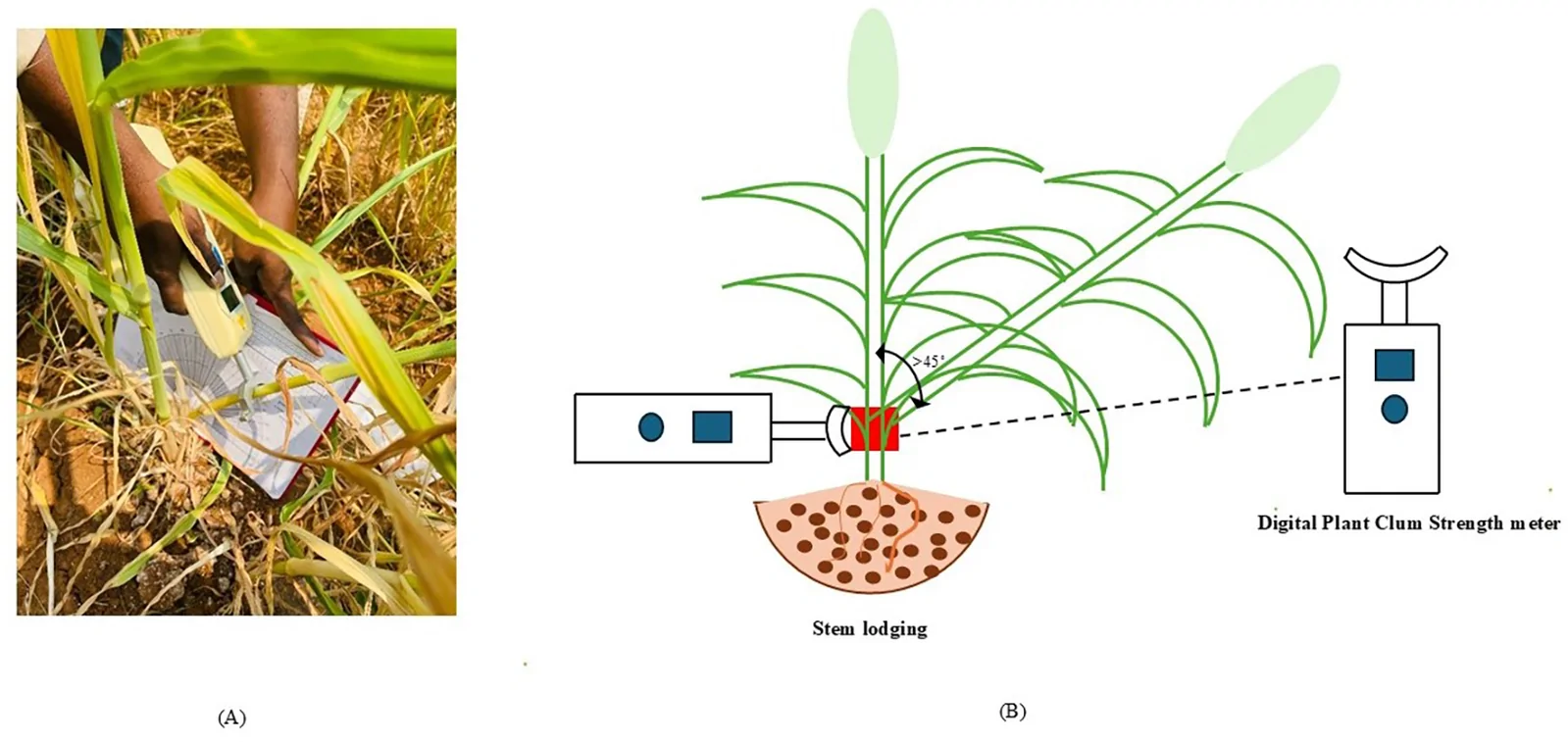
**(A)** A digital plant stem strength meter is used to measure plant culm strength (PCS) in pearl millet. The instrument is placed perpendicular to the main stem at a height of 15 cm above the soil surface and pushed down to the right to an angle of 45° with respect to the vertical to measure pressure in kilopascals (kPa). **(B)** Pictorial presentation of the digital plant culm strength meter used to measure plant culm strength in pearl millet.

### 2.3 Identification of optimal environment for lodging tolerance screening

#### 2.3.1 Experiment II: summer season of 2017

A total of seven hybrid entries (one lodging-susceptible hybrid entry identified from Experiment I and three new lodging-susceptible and three new lodging-tolerant hybrid entries procured from seed companies) were evaluated for plant culm strength and other agro-morphological traits at anthesis, milky-grain, and grain-maturity stages under well-watered (WW) and water-stressed (WS; no irrigation after grain set) conditions during the summer season of 2017. The trial plot consisted of six rows, each 4 m long, with rows spaced 60 cm apart. The experimental plots were fertilized as mentioned in section 2.2.1. The traits like plant culm strength, internode length, culm thickness, culm wall width (**Figure 2A**), dry weight of main stem, dry weight of tillers, and crown root angle (CRA) were measured at anthesis, milky-grain, and grain-maturity stages. In addition, the dry weight of the main stem per tiller shoot (DWMS/tiller shoot), PH per internode length weight (PH/INL weight), and PH per stem weight (PH/stem weight) were estimated across all stages. Further, at the grain-maturity stage, PG was measured under well-watered and water-stressed conditions.

**Figure 2:**
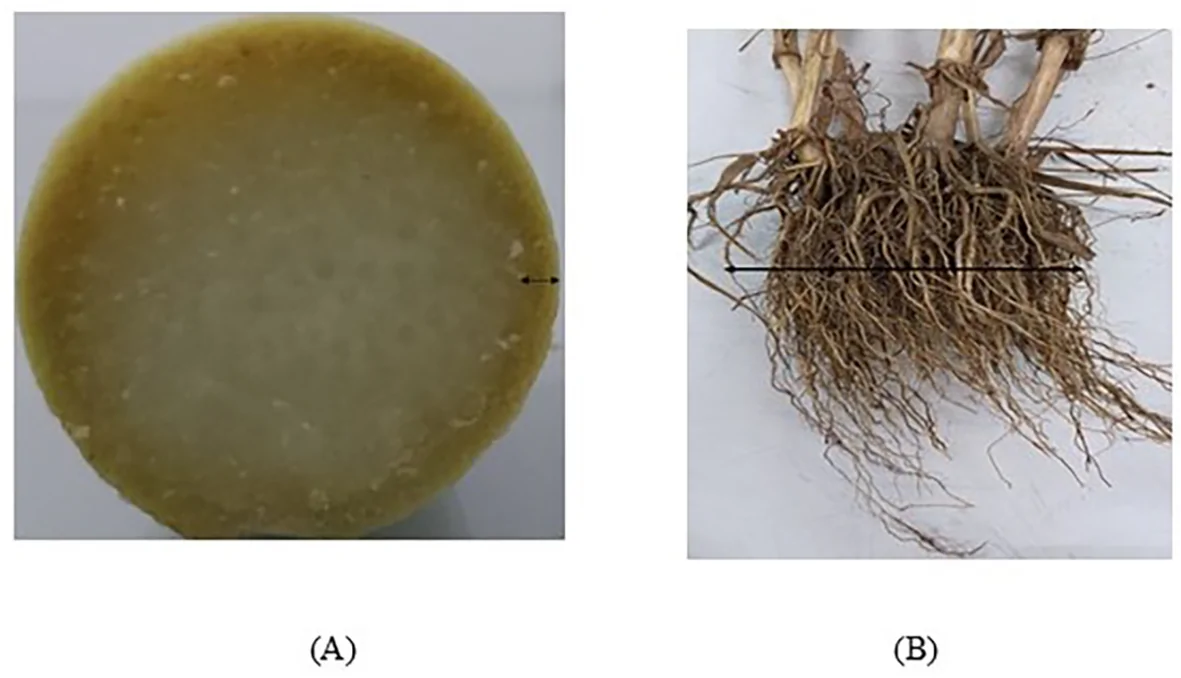
Measurement of lodging-resistance-linked traits: **(A)** culm wall width (CWW) and **(B)** root plate spread (RPS).

### 2.4 Identification of different plant growth stages associated with lodging

#### 2.4.1 Experiment III: rainy season of 2018

A set of 13 hybrid entries (6 lodging-tolerant and 4 lodging-susceptible hybrid entries from Experiments I and II, and 3 new lodging-tolerant hybrid entries, as reported by seed companies) were evaluated to estimate Plant culm strength traits during the rainy season of 2018. The trial was planted in a completely randomized block design with two replications. The trial plot consisted of two rows, each 4 m long, with rows spaced 75 cm apart. The experimental plots were fertilized as mentioned in section 2.2.1. plant culm strength (PCS) was measured at two plant stages, milky-grain and grain-maturity, as described in section 2.2.1.

#### 2.4.2 Experiment IV: summer season of 2019

A total of eight (two lodging-susceptible and six lodging-tolerant) hybrid entries were screened for plant culm strength and other agro-morphological and root traits under well-watered conditions at three plant stages: anthesis, milky-grain, and grain-maturity/harvesting stages during the summer season of 2019. The trial was laid out in a completely randomized block design with two replications. The trial plot consisted of two rows, each 4 m long, with rows spaced 60 cm apart. The experimental plots were fertilized as described in section 2.2.1. The plant culm strength (PCS) was the only trait measured at the anthesis and milky-grain stages. At the grain-maturity stage, traits like PH, NT, INL (between the first and second internodes and between the second and third internodes), CD (between the first and second internodes and between the second and third internodes), PCS, PL, PG, and CR were measured as described in Experiment I. In addition, Lodging score scale (LSS) was recorded visually using a 0–4 scale (0 = no lodging, 4 = severe lodging) at the grain-maturity stage (**Table 2**) (Tian et al., 2010). Furthermore, these randomly selected plants were then excavated from the field using standard shovels without damaging the root system. The excavated plants were shaken for a few minutes to remove soil adhering to the root system. Most of the remaining soil particles were removed by washing the roots at low pressure. After cleaning, the basal stem was cut at ground level, and above-ground traits such as fresh shoot biomass (FSB, g) were measured, while cleaned roots were used to measure fresh root biomass (FRB, g), the horizontal spread of the roots where the majority of rigid root portions terminated, termed root plate spread (RPS, cm) (**Figure 2B**), and crown root angle (CRA) using a protractor scale. Both shoot and root biomass samples were sun-dried for 15 to 20 days, after which dry shoot biomass (DSB, g) and dry root biomass (DRB, g) were measured.

**Table 2:** Lodging score scale (LSS) 0–4 used to evaluate lodging tolerance in pearl millet.

Scale	Description
0	No lodging or slight lean but resuming upright
1	Angle of a plant ≥60˚ and lodged plants <15%
2	Angle of a plant 45 to 60˚ and lodged plants <30%
3	Angle of a plant 30 to 45˚ and lodged plants <50%
4	Angle of a plant <30˚ and lodged plants >50%

Lodging score scale adapted from Tian et al. (2010).

#### 2.4.3 Experiment V: rainy season of 2019

A total of 19 hybrid entries, including 1 susceptible check, 5 tolerant entries, and 13 pipeline hybrid entries (known to be tolerant and procured from eight seed companies), were evaluated during the rainy season of 2019 for Plant culm strength and other agro-morphological traits under well-watered conditions at three stages: anthesis, milky-grain, and grain-maturity/harvesting. The trial was laid out in a completely randomized block design with two replications. The trial plot consisted of two rows, each 4 m long, with rows spaced 75 cm apart. The experimental plots were fertilized as described in section 2.2.1. plant culm strength (PCS) was the only trait measured at the anthesis and milky-grain stages. At the grain-maturity stage, traits like PH, NT, INL, CD, PCS, and CR were measured as described in section 2.2.1, and PL, PG, FSB, FRB, RPS, CRA, DSB, and DRB were measured as described in section 2.4.2.

## 3 Statistical analysis

Analysis of variance (ANOVA) was performed using GenStat^®^ 18th edition (VSN International Limited, Hemel Hempstead, UK) for the experiments described above. Data from different plant stages (anthesis, milky-grain, and grain-maturity) and years were analyzed separately. The means of the traits in the lodging-susceptible and lodging-tolerant groups were compared using the independent t-test or unpaired t-test in GraphPad Prism 5.0 (GraphPad Software, Inc., San Diego, CA, USA). For the rainy season of 2019 data, Tukey’s honestly significant difference (HSD) test was used to identify significant differences between the hybrid entries using GraphPad Prism 5.0. The mean data for agro-morphological and biochemical traits were used to calculate simple Pearson’s correlation coefficients using GraphPad Prism 5.0 (GraphPad Software, Inc., San Diego, CA, USA). Principal component analysis was performed using XLSTAT.

## 4 Results and discussion

### 4.1 Identification of traits affecting lodging

Wide variability was observed for the traits expected to be linked to the lodging-susceptible and lodging-tolerant hybrid entries under study.

#### 4.1.1 Agro-morphological traits

##### 4.1.1.1 Above-ground traits

The overall mean differences in the dry weight of the panicle from the main stem and vegetative tiller number between susceptible and tolerant entries were significant (*P ≤* 0.05), with lodging-tolerant entries having higher values at the milky-grain stage during the rainy season of 2016 (Experiment I) (**Table 3**). At the grain-maturity stage, significant mean differences between susceptible and tolerant entries were found for plant culm strength (PCS; *P ≤* 0.05), PH/culm weight (*P ≤* 0.01), panicle girth (PG; *P ≤* 0.05), and lodging percentage (*P ≤* 0.05) (**Table 4**). Numerous studies have shown that the plant culm strength trait measured using a digital plant culm strength meter clearly differentiates lodging-tolerant entries from lodging-susceptible entries in other crops (Gu et al., 2017; Xue et al., 2020; Yang et al., 2022). For panicle girth, earlier studies have shown that panicle traits such as panicle girth and compact panicle can impact lodging incidence in various crops (Xu et al., 2005; Wu et al., 2011; Blösch et al., 2020). The lodging-tolerant entries had a significantly lower PH/culm weight ratio than the lodging-susceptible entries, indicating that the lodging-tolerant entries had greater or similar PH and higher culm weight than the lodging-susceptible entries. These findings were in agreement with the observations of Rao et al. (2017), who reported that lodging-tolerant entries showed greater plant height and culm length than lodging-susceptible entries in rice. Overall, the PCS, PH/culm weight, and PG traits clearly differentiated the lodging-tolerant and lodging-susceptible entries at the grain-maturity stage.

**Table 3:** Lodging-tolerance-associated traits in pearl millet evaluated at anthesis and milky-grain stages during the rainy season of 2016 (Experiment I) at ICRISAT, Patancheru.

Anthesis																						
Entry	Lodging reactions	PH (cm)	VT	PT	CD (cm)	CRA (°)	CRN	DWMS/tiller shoot	DW of leaf of MS	DW of leaf of tiller	DW of panicle of MS	DW of stem of MS	DW of stem of tiller	TDW of MS	TDW of Tiller	PH/stem weight	ADL of MS (%)	HC of MS (%)	Cellulose of MS (%)	ADL of PT (%)	HC of PT (%)	Cellulose of PT (%)
PMCHT01	Tolerant	169.83	4.4	NA	11.95	63.75	38.1	7.65	6.12	6.32	10.81	32.7	3.41	49.62	17.11	5.53	4.99	19.23	35.23	3.66	20.92	36.54
PMCHT02	Tolerant	179.2	4.7	NA	13.21	65	51.9	7.23	6.81	7.65	12.58	38.86	9.75	53.49	9.34	5.27	4.82	19.08	35.02	3.62	22.06	35.21
PMCHT03	Tolerant	176.7	6.3	NA	13.07	64.85	52	2.78	7.89	11.9	11.36	39.09	11.78	54.85	19.09	4.73	4.93	NA	35.43	4.28	19.95	40.84
PMCHT04	Tolerant	183	4.6	NA	13.51	63.85	44.4	6.29	7.25	7.77	9.5	34.82	3.11	51.57	8.97	5.51	4.92	19.12	35.53	NA	NA	NA
Average		177.18	5.00	NA	12.94	64.36	46.60	5.99	7.02	8.41	11.06	36.37	7.01	52.38	13.63	5.26	4.92	19.14	35.30	3.85	20.98	37.53
PMCHT11	Susceptible	148.05	4.2	NA	11.56	61.3	45.8	3.78	4.19	6.98	8.27	28.67	10.07	41.13	13.65	5.75	4.76	19.1	34.31	3.81	20.87	34.35
PMCHT12	Susceptible	157.15	4.7	NA	11.52	60.75	47.8	3.43	4.45	8.49	11.43	20.95	3.07	36.83	11.56	7.96	5.01	18.49	33.7	3.79	20.9	35.27
PMCHT13	Susceptible	198.62	5	NA	14.82	58.85	43.2	7.54	8.86	8.33	9.97	38.38	6.45	57.21	12.35	5.55	4.72	20.89	35.17	NA	NA	NA
Average		167.94	4.63	NA	12.63	60.30	45.60	4.92	5.83	7.93	9.89	29.33	6.53	45.06	12.52	6.42	4.83	19.49	34.39	3.80	20.89	34.81
t-test		0.58	0.74	NA	0.26	5.01*	0.28	0.62	0.76	0.37	1.05	1.33	0.16	1.16	0.41	1.46	0.87	0.49	2.06	0.25	0.15	1.55
p-value		0.62	0.50	NA	0.82	0.04	0.80	0.57	0.53	0.74	0.37	0.31	0.88	0.36	0.71	0.28	0.47	0.68	0.18	0.83	0.89	0.26
Critical Difference		28.2	2.42	NA	2.21	9.21	21.52	3.88	2.66	4.84	4.67	25.3	2.49	26.34	8.03	3.39	0.21	1.42	2.11	0.10	1.90	0.17
Coefficient of Variation (%)		4.7	14.4	NA	5	4.2	13.5	20.3	11.9	17.1	12.8	21.9	10.6	15.5	17.6	17.0	1.2	2.1	1.7	0.7	2.6	0.1
Milky-grain																						
PMCHT01	Tolerant	164.5	4.1	1.75	11.15	50.75	40.4	9.98	5.34	5.25	30.87	16.91	3.57	50.83	4.9	10.38	5.69	16.64	37.49	4.93	19.9	28.64
PMCHT02	Tolerant	180.2	4.1	2.25	11.55	51.25	56.6	9.36	6.71	6.19	33.99	19.77	2.56	62.66	8.74	8.97	5.86	16.49	37.68	5.18	20.12	31.2
PMCHT03	Tolerant	173.8	4.7	1.25	11.62	55	43.5	9.02	5.7	6.24	30.1	17.16	2.65	52.97	7.98	10.34	5.52	16.54	36.5	4.89	19.24	29.03
PMCHT04	Tolerant	176.9	3.8	1.00	11.8	51.5	38.8	11.4	6.15	3.81	27.75	17.93	1.25	51.84	5.06	10.35	5.77	16.38	36.99	5.6	20.14	30.78
Average		173.85	4.18	1.56	11.53	52.13	44.83	9.94	5.98	5.37	30.68	17.94	2.51	54.58	6.67	10.01	5.71	16.51	37.17	5.15	19.85	29.91
PMCHT11	Susceptible	144.03	3.8	1.00	9.48	51.25	53	5.54	3.15	5	20.95	10.61	2.68	34.71	7.58	14.4	5.52	17.26	35.86	5.5	19.03	32.04
PMCHT12	Susceptible	160.8	3.4	1.50	10.35	49.25	38.2	8.4	3.67	3.4	21.65	11.74	1.46	37.06	4.86	14.15	5.65	16.69	36.03	5.19	19.04	30.05
PMCHT13	Susceptible	197.2	3.3	1.42	13.34	49.75	37.7	16.69	7.4	3.35	26.41	21.07	1.7	54.88	5.06	10.51	5.38	17.7	34.82	NA	NA	NA
Average		167.34	3.50	1.31	11.06	50.08	42.97	10.21	4.74	3.92	23.00	14.47	1.95	42.22	5.83	13.02	5.52	17.22	35.57	5.35	19.04	31.05
t-test		0.41	2.78*	0.81	0.40	1.79	0.29	0.08	0.90	1.85	3.58*	1.03	0.93	1.78	0.63	2.31	1.82	2.79*	3.45*	0.87	3.87*	0.96
p-value		0.72	0.05	0.47	0.73	0.15	0.79	0.94	0.46	0.14	0.02	0.41	0.41	0.22	0.56	0.15	0.14	0.04	0.04	0.45	0.03	0.51
Critical Difference at 5%		22.56	2.46	0.83	5.74	23.43	9.83	1.45	4.22	16.06	9.34	1.49	29.48	2.46	3.81	0.52	3.5	3.5	2.73	0.17	0.38	0.73
Coefficient of Variation (%)		3.8	18.3	16.8	3.9	3.2	15.4	28.3	7.7	25.7	17.0	16.4	19.1	17.3	17.9	9.7	2.7	6.0	2.2	1.0	0.6	0.7

PH, Plant height; VT, Vegetative tiller; PT, Productive tiller; CD, Culm diameter; CRA, Crown root angle; CRN, Crown root number; DWMS/tiller shoot, Dry weight of main stem per tiller shoot; DW of leaf of MS, Dry weight of leaf of main stem; DW of leaf of tiller, Dry weight of leaf of tiller; DW of panicle of MS, Dry weight of panicle of main stem; DW of stem of MS, Dry weight of stem of main stem; DW of stem of tiller, Dry weight of stem of tiller; TDW of MS, Total dry weight of main stem; TDW of tiller, Total dry weight of tiller; PH/stem weight, Plant height per stem weight; ADL of MS, Acid detergent lignin of main stem; HC of MS, Hemicellulose of main stem; Cellulose of MS, Cellulose of main stem; ADL of PT, Acid detergent lignin of productive tillers; HC of PT, Hemicellulose of productive tiller; Cellulose of PT, Cellulose of productive tiller. **P ≤ 0.01; *P ≤ 0.05.

**Table 4:** Lodging-tolerance-associated traits in pearl millet evaluated at the grain-maturity stage during the rainy season of 2016 (Experiment I) at ICRISAT, Patancheru.

Grain-maturity																									
Entry	Lodging reactions	PCS (kPa)	PH (cm)	PL (cm)	PG (mm)	DW of leaf of MS	DW of stem of MS	DW of panicle of MS	DW of leaf tillers	DW of stem of tillers	DW of leaf PT	DW of stem of PT	DW of panicle of PT	TDW of MS	TDW of tillers	TDW of PT	DWMS/tiller shoot	PH/stem weight	Lodging (%)	ADL of MS (%)	HC of MS (%)	Cellulose of MS (%)	ADL of PT (%)	HC of PT (%)	Cellulose of PT (%)
PMCHT01	Tolerant	265	181.6	23.16	32.75	4.38	23.16	26.83	3.81	1.03	0.96	2.49	1.64	54.38	4.24	5.1	12.86	7.84	5.0	6.72	10.38	43.04	5.83	16.53	32.36
PMCHT02	Tolerant	300	195.9	26.95	31.64	3.66	20.38	19.95	2.47	1.17	0.55	1.54	1.04	43.99	3.04	3.13	14.49	9.72	5.0	6.43	12.11	41.64	5.38	17.49	31.27
PMCHT03	Tolerant	330	204.1	24.42	30.75	4.91	23.56	27.14	6.9	2.36	0.7	2.87	3.17	55.61	10.84	6.75	5.27	8.64	1.83	6.91	11.91	41.18	6.29	15.65	31.97
PMCHT04	Tolerant	350	200.1	24.68	31.68	6.06	31.37	31.6	5.76	0.73	1.18	4.33	3.42	69.03	6.49	8.92	11.13	6.5	5.0	6.25	7.06	38.36	NA	NA	NA
Average		311	195.4	24.80	31.71	4.75	24.62	26.38	4.74	1.32	0.85	2.81	2.32	55.75	6.15	5.98	10.94	8.18	4.21	6.58	10.37	41.06	5.83	16.56	31.87
PMCHT11	Susceptible	340	181.1	21.65	26.92	2.68	13.45	18.83	3.94	1.15	1.84	8.81	7.22	34.96	4.4	20.4	12.17	13.47	7.0	7.03	12.61	43.98	6.39	16.27	33.44
PMCHT12	Susceptible	220	196.8	20.96	26.86	3.16	13.7	16.37	3.69	1	2.56	8.77	5.08	33.22	9.33	16.18	5.07	15.24	6.5	7.26	10.45	41.27	6.5	14.46	34.47
PMCHT13	Susceptible	220	207.9	25.45	28.15	5.51	22.75	26.06	1.96	1.03	0.44	1.49	0.93	54.32	2.38	3.02	24.41	9.18	6.5	6.42	13.94	41.35	5.46	17.57	29.36
Average		260	195.3	22.69	27.31	3.78	16.63	20.42	3.20	1.06	1.61	6.36	4.41	40.83	5.37	13.20	13.88	12.63	6.67	6.90	12.33	42.20	6.12	16.10	32.42
t-test		4.02*	0.02	1.32	7.42**	0.96	2.07	1.58	1.32	0.73	1.20	1.42	1.08	1.76	0.29	1.34	0.49	2.61*	2.59*	1.12	1.27	0.86	0.67	0.44	0.35
p-value		0.01	0.99	0.28	0.00	0.41	0.11	0.19	0.26	0.52	0.35	0.29	0.39	0.15	0.79	0.31	0.67	0.05	0.05	0.34	0.27	0.44	0.55	0.69	0.76
Critical Difference at 5%		184.1	39.87	3.95	4.01	2.25	9.52	14.71	2.18	0.31	0.66	2.11	2.11	22.39	2.35	7.96	12.11	7.47	4.60	2.53	5.92	13.60	0.97	1.66	2.60
Coefficient of Variation (%)		20.3	5.9	4.8	3.9	15.0	13.0	17.8	15.6	7.5	16.0	14.1	18.9	13.11	11.7	25.4	28.7	21.4	25.3	10.9	15.3	9.5	4.7	3.0	2.3

PCS, Plant culm strength; PH, Plant height; PL, Panicle length; PG, Panicle girth; DW of leaf of MS, Dry weight of leaf of main stem; DW of stem of MS, Dry weight of stem of main stem; DW of panicle of MS, Dry weight of panicle of main stem; DW of leaf of tillers, Dry weight of leaf of tillers; DW of stem of tillers, Dry weight of stem of tillers; DW of stem of PT, Dry weight of stem of productive tiller; DW of panicle of PT, Dry weight of panicle of productive tiller; TDW of MS, Total dry weight of main stem; TDW of tillers, Total dry weight of tillers; TDW of PT, Total dry weight of productive tiller; DWMS/tiller shoot, Dry weight of main stem per tiller shoot; PH/stem weight, Plant height per stem weight; ADL of MS, Acid detergent lignin of main stem; HC of MS, Hemicellulose of main stem; Cellulose of MS, Cellulose of main stem; ADL of PT, Acid detergent lignin of productive tillers; HC of PT, Hemicellulose of productive tiller; Cellulose of PT, Cellulose of productive tiller. **P ≤ 0.01; *P ≤ 0.05.

During the rainy season of 2018 (Experiment III), the mean PCS values of tolerant hybrid entries (390 kPa; 523 kPa) were significantly higher than those of susceptible hybrid entries (277 kPa; 341 kPa) at the milky-grain (*P ≤* 0.001) and grain-maturity (P ≤ 0.05) stages (**Table 5**). In the summer season of 2019, the overall mean values of culm diameter (CD1-2, *P ≤* 0.05; CD2-3, *P* ≤ 0.05) in tolerant hybrid entries were significantly higher than those in susceptible hybrid entries at the grain-maturity stage (**Table 6**). Earlier studies have reported that increasing the culm diameter of the basal internodes is one of the key steps in increasing culm bending resistance, thereby reducing the chance of lodging (Kashiwagi et al., 2008; Hirano et al., 2014; Piñera-Chavez et al., 2016; Yao et al., 2011). In addition, Borale et al. (2007) found that higher culm diameter was linked to lodging resistance among 10 promising entries tested in an advanced varietal trial of pearl millet. Again, at grain-maturity, the mean Lodging score scale (LSS) of lodging-tolerant hybrid entries (1.33) was significantly different (*P* ≤ 0.01) from that of susceptible hybrid entries (2.50) during the summer season of 2019, consistent with the existing classification of these hybrid entries based on their lodging reaction.

**Table 5:** Lodging-tolerance-associated traits in pearl millet evaluated during the rainy season of 2018 (Experiment III) at ICRISAT, Patancheru.

Genotypic code	Lodging reactions	PCS (kPa)	
		Milky-grain	Grain-maturity
PMCHT04	Tolerant	368	501
PMCHT02	Tolerant	438	590
PMCHT03	Tolerant	414	517
PMCHT05	Tolerant	487	600
PMCHT06	Tolerant	325	479
PMCHT07	Tolerant	454	655
PMCHT08	Tolerant	350	602
PMCHT09	Tolerant	308	367
PMCHT10	Tolerant	369	400
Average		390.33	523.44
PMCHT11	Susceptible	265	248
PMCHT12	Susceptible	253	213
PMCHT15	Susceptible	297	380
PMCHT16	Susceptible	294	524
Average		277.3	341.3
t-test		4.90***	2.73*
p-value		0.001	0.02
Critical difference at 5%		198.14	269.02
Coefficient of variation (CV, %)		18.11	18.0

PCS, Plant culm strength. ***P ≤ 0.001; **P ≤ 0.01; *P ≤ 0.05.

**Table 6:** Lodging-tolerance-associated traits in pearl millet evaluated during the summer season of 2019 (Experiment IV) at ICRISAT, Patancheru.

Genotypic code	Lodging reactions	Anthesis	Milky-grain	Grain-maturity																
		PCS (kPa)	PCS (kPa)	PCS (kPa)	PH (cm)	NT	CD_1_2	CD_2-3	INL_1_2 (cm)	INL_2-3 (cm)	PL (cm)	PG (mm)	CRA (°)	RPS (cm)	FSB (g)	DSB (g)	FRB (g)	DRB (g)	GY (g/plant)	LSS
PMCHT08	Tolerant	202.00	209.20	294.70	147.65	2.55	10.43	10.41	15.8	17.65	27.78	21.95	58.72	7.52	114.55	0.47	14.9	0.38	17.22	0.5
PMCHT05	Tolerant	139.20	164.50	267.70	153	2.3	11.01	10.99	13.55	15.8	28.61	24.45	56.95	7.3	100.8	0.55	9.7	0.39	10.52	1
PMCHT07	Tolerant	159.20	167.50	240.90	157.1	4	11.2	11.18	15.7	16.65	29.44	25.3	54.3	8.35	108.8	0.47	14.6	0.45	9.67	1
PMCHT10	Tolerant	168.15	190.65	250.90	150.9	2.7	10.49	11.44	16.68	17.8	27.01	26.2	55.25	8.07	109.9	0.53	16.7	0.53	15.90	1.5
PMCHT01	Tolerant	195.00	210.30	236.40	138.9	2.3	10.15	10.65	14.85	15.75	29.54	23	57.55	7.8	83.5	0.4	14.6	0.44	7.17	2
PMCHT02	Tolerant	250.67	258.10	326.25	154.2	3	11.05	11.15	16.23	16.05	30.97	26.05	58.35	8.55	120.2	0.59	22.05	0.46	15.90	2
Average		185.70	200.04	269.48	150.29	2.81	10.72	10.97	15.47	16.62	28.89	24.49	56.85	7.93	106.29	0.50	15.43	0.44	12.73	1.33
PMCHT11	Susceptible	141.30	159.75	173.80	142.2	4.5	9.71	9.6	13.9	15.65	26.23	20.55	55.5	6.88	78.35	0.48	10.55	0.38	14.33	2.5
PMCHT16	Susceptible	117.40	141.60	196.70	144.4	4.2	9.41	10.14	13.9	15.4	26.68	25.13	58.1	7.7	83.2	0.45	7.6	0.38	12.37	2.5
Average		129.35	150.68	185.25	143.30	4.35	9.56	9.87	13.90	15.53	26.46	22.84	56.80	7.29	80.78	0.47	9.08	0.38	13.35	2.50
t-test		2.81*	2.94	4.60*	2.46	5.11*	5.09*	3.66*	3.44*	2.77*	3.95*	0.69	0.04	1.41	4.40**	1.16	2.89	2.85*	0.32	4.60**
p-value		0.05	0.03	0.01	0.06	0.004	0.015	0.01	0.02	0.04	0.01	0.62	0.98	0.39	0.01	0.30	0.06	0.04	0.76	0.01
Critical difference at 5%		84.55	114.22	128.26	17.43	3.24	2.61	2.71	2.68	4.41	2.91	4.05	11.67	2.68	44.55	0.33	9.43	0.43	11.81	2.7
Coefficient of variation (CV, %)		14.9	18.4	16.0	3.5	29.7	7.6	7.7	5.4	8.2	3.1	5.1	6.1	10.4	13.5	20.3	21.7	30.1	26.3	2.9

PCS, Plant culm strength; PH, Plant height; NT, Number of tillers; CD, Culm diameter; INL, Internode length; PL, Panicle length; PG, Panicle girth; CRA, Crown root angle; RPS, Root plate spread; FSB, Fresh shoot biomass; DSB, Dry shoot biomass; FRB, Fresh root biomass; DRB, Dry root biomass; LSS, Lodging score scale; GY, Grain yield. **P ≤ 0.01; *P ≤ 0.05.

During the summer season of 2019 (Experiment IV), the overall mean number of tillers in tolerant hybrid entries was significantly lower (*P* ≤ 0.05) than that in susceptible hybrid entries at the grain-maturity stage (**Table 6**), indicating that reduced tiller number must be considered when breeding lodging-tolerant hybrid entries. It has also been reported that increased CD reduces tiller number (Tripathi et al., 2003), as found in this study. This is in agreement with earlier findings from other crops (Keller et al., 1999; Tripathi et al., 2003). The overall mean internode lengths (INL1-2, *P* ≤ 0.05; INL2-3, *P* ≤ 0.05) were greater in lodging-tolerant entries than in lodging-susceptible entries. A study by Tripathi et al. (2003) identified increased culm diameter, thicker internodes, and fewer tillers per plant as important traits for studying lodging tolerance in spring wheat. The mean panicle length of lodging-tolerant entries was significantly higher (*P* ≤ 0.01) than that of lodging-susceptible entries. These findings are in accordance with previous work by Assefa et al. (2002) and Girma (2019), who showed that panicle length had a strong positive correlation with lodging index in teff. The overall mean fresh shoot biomass (FSB) of tolerant hybrid entries was significantly higher (*P* ≤ 0.01) than that of susceptible hybrid entries at the grain-maturity stage during the summer season of 2019 (Experiment IV) (**Table 6**), indicating that stronger culms support higher fresh shoot biomass accumulation. This finding corroborates the result reported by Ding et al. (2025) in maize. Overall, PCS (across crop growth stages) and the CD, tiller number, internode length, panicle length, and fresh shoot biomass traits (at the grain-maturity stage) differed greatly between lodging-tolerant and lodging-susceptible entries.

In Experiment V (rainy season of 2019), one, four, and five known lodging-tolerant hybrids and two, five, and five pipeline hybrids had significantly greater PCS values than the lodging-susceptible check entry PMCHT11 at the anthesis, milky-grain, and grain-maturity stages, respectively (**Table 7**). Again, at grain-maturity, the known lodging-tolerant entry PMCHT08 for INL (between the first and second nodes) and PMCHT06 and PMCHT08 for INL (between the second and third nodes) were significantly different from the lodging-susceptible check entry PMCHT11 during the rainy season of 2019 (**Table 7**). Earlier studies have also suggested that desirable internode length is an important trait in breeding cultivars for lodging tolerance in rice and wheat (Islam et al., 2007; Kong et al., 2013; Kuai et al., 2015). For PL, one known lodging-tolerant entry (PMCHT07) and one pipeline hybrid entry (PMCHT20) were significantly different from the lodging-susceptible check entry PMCHT11 at the grain-maturity stage during the rainy season of 2019. The study by Yadav et al. (2017) reported that panicle length was positively and significantly associated with lodging tolerance in rice. Furthermore, for PG, 4 of 5 known lodging-tolerant hybrid entries and 9 of 13 pipeline hybrid entries were significantly different from the lodging-susceptible check entry PMCHT11 at grain-maturity during the rainy season of 2019 (**Table 7**). Overall, PCS clearly differentiated lodging-tolerant and lodging-susceptible entries at the milky-grain and grain-maturity stages. Also, the PG of lodging tolerant entries and pipeline hybrid entries showed greater significant differences than lodging susceptible entries.

**Table 7:** Lodging-tolerance-associated traits in pearl millet evaluated during the rainy season of 2019 (Experiment V) at ICRISAT, Patancheru.

Genotypic code	Lodging reaction	Anthesis		Milky-grain	Grain-maturity													
		DB	PCS (kPa)	PCS (kPa)	PCS (kPa)	PH (cm)	NT	CD 1-2	CD (2-3)	INL (1-2)	INL (2-3)	PL (cm)	PG (mm)	LSS	FSB (g)	RPS (cm)	CRA	FRB (g)
PMCHT05	Tolerant	51^ef^	415.7^ab^	530.5^cd^	694.9^bc^	257^def^	2.9^a^	9.40^a^	10.41^a^	20.8^ab^	23.1^abc^	24.6^abc^	33.36^bc^	1^a^	237.3^a^	6.7^a^	54.8^a^	25.1^ab^
PMCHT06	Tolerant	51^ef^	395.5^ab^	529.9^cd^	771.8^c^	259^def^	3^a^	10.28^a^	10.62^a^	21.6^ab^	24.1^bc^	23.8^abc^	33.32^bc^	1.5^a^	291.5^a^	6.7^a^	57.3^a^	42.9^ab^
PMCHT07	Tolerant	52^f^	436.2^abc^	568.3^d^	636.6^bc^	274^f^	2.9^a^	9.16^a^	9.93^a^	20.6^ab^	23.8^abc^	26.6^c^	32.34^bc^	2.5^a^	256.2^a^	8.1^a^	52.3^a^	33.0^ab^
PMCHT08	Tolerant	48^abc^	443.5^abc^	565.5^d^	686.3^bc^	242^bcde^	3.1^a^	9.14a	10.39^a^	22.5^b^	24.4^c^	22.3^abc^	32.46^bc^	0^a^	220.5^a^	7.2^a^	57.8^a^	47.4^ab^
PMCHT10	Tolerant	50.5^def^	696.9^c^	472.8^bcd^	652.4^bc^	234.5^abcd^	2.8^a^	8.62^a^	9.43^a^	21.1^ab^	22.8^abc^	23.8^abc^	31.08^ab^	1^a^	254.5^a^	6.9^a^	56.5^a^	38.0^ab^
PMCHT17	Expected to be tolerant	51^ef^	477.1^abc^	530.5^cd^	641b^c^	266^ef^	2.9^a^	9.10^a^	9.41^a^	21.4^ab^	23.3^abc^	22.8^abc^	31.06^ab^	2^a^	270.7^a^	7.5^a^	52.5^a^	36.6^ab^
PMCHT18	Expected to be tolerant	48.5^bcd^	423^ab^	371.3^abcd^	518^abc^	240^bcde^	3.3^a^	9.02a	10.29^a^	19.5^ab^	22.2^abc^	24.8^abc^	35.29^bc^	2^a^	203.0^a^	6.1^a^	55.5^a^	54.0^b^
PMCHT19	Expected to be tolerant	46^a^	378.6^ab^	500.3^cd^	476.7^ab^	239^bcde^	3.3^a^	7.49a	8.36^a^	20.4^ab^	23.1^abc^	23.0^abc^	31.69^ab^	2.5^a^	178.9^a^	7.1^a^	52.8^a^	24.2^ab^
PMCHT20	Expected to be tolerant	48^abc^	344.3^ab^	411.6^abcd^	529.7^abc^	233^abcd^	3.1^a^	8.88a	9.62^a^	20.2^ab^	22.3^abc^	25.2^bc^	34.85^bc^	1^a^	202.7^a^	7.3^a^	53.5^a^	32.1^ab^
PMCHT21	Expected to be tolerant	48^abc^	475.3^abc^	385.5^abcd^	590.4^bc^	226^abc^	3.1^a^	9.91a	10.17^a^	21.9^ab^	22.8^abc^	20.2^ab^	31.18^ab^	1.5^a^	183.1^a^	6.9^a^	51.0^a^	32.9^ab^
PMCHT22	Expected to be tolerant	47^ab^	340.6^ab^	445.7^abcd^	507.8^abc^	229^abcd^	2.9^a^	9.79a	9.95^a^	20.4^ab^	22.6^abc^	23.7^abc^	33.11^bc^	1^a^	238.3^a^	7.5^a^	51.4^a^	35.0^ab^
PMCHT23	Expected to be tolerant	48^abc^	319^ab^	429.1^abcd^	497^abc^	232^abcd^	3.3^a^	8.53a	9.23^a^	20.9^ab^	23.4^abc^	22.1^abc^	35.00^bc^	4^a^	163.4^a^	7.1^a^	54.5^a^	28.1^ab^
PMCHT24	Expected to be tolerant	46^a^	376.1^ab^	332.8^abc^	576.4^abc^	244^bcdef^	2.9^a^	8.34a	9.12^a^	20.6^ab^	22.4^abc^	23.6^abc^	33.19^bc^	1^a^	229.3^a^	8.2^a^	51.8^a^	48.2^ab^
PMCHT25	Expected to be tolerant	49.5^cde^	388^ab^	577.8^d^	519.3^abc^	244^bcdef^	3.5^a^	10.82^a^	11.3^a^	20.5^ab^	22.7^abc^	23.6^abc^	31.51^ab^	2^a^	199.4^a^	9.1^a^	49.5^a^	24.3^ab^
PMCHT26	Expected to be tolerant	48.5^bcd^	576^bc^	521.4^cd^	676.2^bc^	223^ab^	3.3^a^	10.22a	10.52^a^	20.2^ab^	22.0^abc^	24.3^abc^	35.32^bc^	1.5^a^	279.4^a^	7.5^a^	49.0^a^	35.6^ab^
PMCHT27	Expected to be tolerant	46^a^	352.6^ab^	241.6^a^	490.2^abc^	218^ab^	3.4^a^	9.47a	9.55^a^	19.2^ab^	21.8^abc^	21.0^ab^	34.39^bc^	3^a^	249.2^a^	7.5^a^	56.3^a^	36.4^ab^
PMCHT28	Expected to be tolerant	51^ef^	460.5^abc^	376.7^abcd^	638.3^bc^	254^cdef^	3.3^a^	10.31^a^	10.64^a^	18.2^ab^	20.5^abc^	24.1^abc^	33.11^bc^	2^a^	217.4^a^	6.5^a^	49.3^a^	26.0^ab^
PMCHT29	Expected to be tolerant	51^ef^	486.5^bc^	531.5^cd^	629.5^bc^	221^ab^	3.2^a^	8.89^a^	9.93^a^	17.3^ab^	19.6^ab^	21.6^abc^	38.69^c^	0^a^	277.7^a^	7.2^a^	56.3^a^	42.2^ab^
PMCHT11	Susceptible	46^a^	210.9^a^	282.4^ab^	295.8^a^	206^a^	2.9^a^	7.31a	8.09^a^	16.7^a^	19.4^a^	19.9^a^	24.95^a^	3.5^a^	136.1^a^	6.4^a^	54.9^a^	18.7^a^
Critical difference at 5%		1.17	195.09	162.97	229.08	22.79	0.86	2.70	2.70	3.77	3.27	2.94	6.12	2.30	0.11	2.56	12.39	0.03
Coefficient of variation (CV, %)		1.2	16.3	12.6	13.8	3.3	9.3	9.9	9.3	6.3	4.9	4.2	6.3	26.3	17.5	12.0	7.8	19.5

DB, Days to 50% bloom; PCS, Plant culm strength; PH, Plant height; NT, Number of tillers; CD, Culm diameter; INL, Internode length; PL, Panicle length; PG, Panicle girth; LSS, Lodging score scale; FSB, Fresh shoot biomass; RPS, Root plate spread; CRA, Crown root angle; FRB, Fresh root biomass. Means followed by different letters indicate significant differences between genotypes.

Across the experiments, our study found that as plant developmental stages advanced, lodging-tolerant hybrid entries showed higher mean PCS values than lodging-susceptible hybrid entries. This was in line with the findings from earlier studies in rice (Luo et al., 2007; Gui et al., 2018) and in wheat (Chen et al., 2011). Overall, the multi-season and multi-year study identified plant culm strength (PCS) across crop growth stages and panicle girth (PG) at the grain-maturity stage as two important indicators of lodging tolerance that could help screen a larger number of breeding materials for lodging tolerance in pearl millet under field conditions.

##### 4.1.1.2 Below-ground traits

In Experiment I (rainy season of 2026), the overall mean CRA of susceptible and tolerant hybrid entries was significant (*P* ≤ 0.05) at the anthesis stage (**Table 3**). The mean CRA of lodging-tolerant hybrid entries was significantly larger than that of lodging-susceptible hybrid entries. Similarly, Zhang et al. (2022) found that lodging-resistant hybrid entries had a wider root angle than lodging-susceptible hybrid entries in maize. During the summer season of 2019 (Experiment IV), the overall mean DRB of lodging-tolerant entries was significantly higher (*P* ≤ 0.05) than that of lodging-susceptible entries (**Table 6**). These results are in agreement with those of Wu et al. (2018) and Bengough et al. (2011), who reported that root dry weight was closely associated with lodging resistance. For FRB, one pipeline entry PMCHT18 was significantly different from the lodging-susceptible check entry PMCHT11 at the grain-maturity stage during the rainy season of 2019 (Experiment V) (**Table 7**), indicating that high root biomass can enhance plant anchoring ability and reduce the risk of lodging. This is consistent with the findings of Liu et al. (2024), who reported that fresh root biomass strengthens plant anchorage under optimum planting density in oat.

##### 4.1.1.3 Biochemical traits

The mean cellulose (*P* ≤ 0.05) and hemicellulose (HC; *P* ≤ 0.05) contents of the main stem and HC content (*P* ≤ 0.05) of the productive tillers differed significantly between susceptible and tolerant hybrid entries at the milky-grain stage during the rainy season of 2016 (Experiment I) (**Table 3**). In this study, significantly higher overall mean values of cellulose and HC in lodging-tolerant hybrid entries than in lodging-susceptible hybrid entries at the milky-grain stage indicated that these biochemical constituents provide mechanical support to the plants. Numerous studies have indicated that lodging-resistant cultivars accumulate more cellulose and hemicellulose than lodging-susceptible cultivars in several other crops (Liu et al., 2016; Peng et al., 2014; Zhang et al., 2020). The overall mean ADL, cellulose, and HC values of lodging-tolerant and lodging-susceptible hybrid entries increased as phenological stages advanced; however, there were no significant differences between the hybrid entries at the anthesis and grain-maturity stages.

#### 4.1.2 Correlation between plant culm strength and agro-morphological and biochemical traits

The PCS value of lodging-tolerant entries was found to be significantly positively correlated with CD (r = 1.00, *p* < 0.01) (**Supplementary Table 3**) at maturity under well-watered conditions in Experiment II, indicating that lodging-tolerant entries with thicker culms strongly support lodging tolerance. This finding is consistent with the reports of Kashiwagi and Ishimaru (2004) and Ookawa et al. (2010), who found a highly significant positive correlation between culm diameter and culm strength traits in rice. Again, in Experiment II, the PCS values of lodging-susceptible entries showed highly significant positive correlations with CD (r = 1.00, *p* < 0.01 and r = 0.98, *p* < 0.01, WW and WS, respectively) and significant negative correlations with CRA (r = -1.00, *p* < 0.01 and r = -0.98, *p* < 0.01, WW and WS, respectively) at the milky-grain stage, indicating that these entries were prone to root lodging. The PCS value of lodging tolerant entries was found to have a significantly negative correlation with NT (r = -0.47, *p* < 0.05) at maturity stage in Experiment-V, indicating that lodging tolerant entries having thicker culm, and thicker culm reduces the tiller numbers. This finding is consistent with previous reports by Tripathi et al. (2003) and Bayable et al. (2020) in teff.

#### 4.1.3 Principal component analysis (PCA)

PCA was carried out for Experiment V because this experiment included lodging-tolerant, lodging-susceptible, and pipeline hybrid (expected to be tolerant) groups. The first two principal components explained 50.93% of the variation, according to the PCA biplot. The first principal component explained 34.7% of the variation, while the second principal component explained 16.18% (**Figure 3**). The PCA biplot clearly differentiated lodging-tolerant and lodging-susceptible entries. For instance, all lodging-tolerant entries grouped together in the upper-right quadrant, while the lodging-susceptible entry was in the upper-left quadrant.

**Figure 3:**
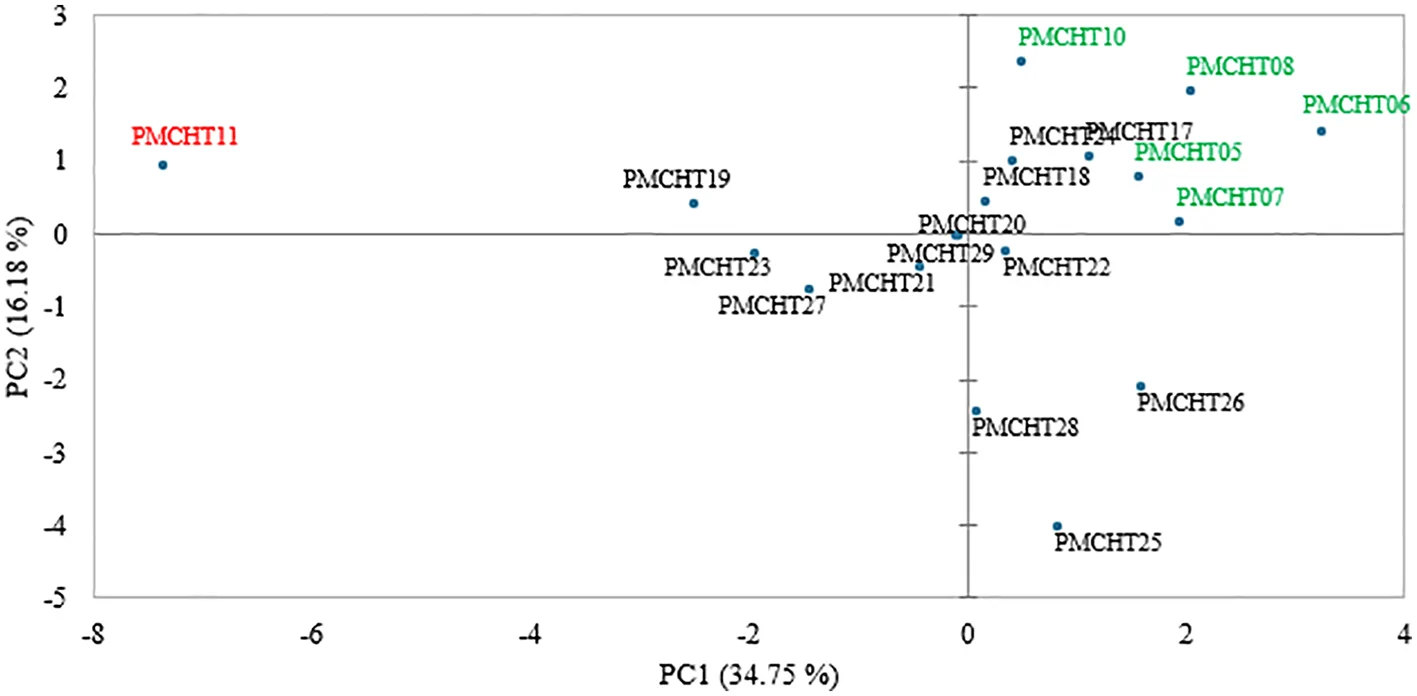
Principal component analysis (PCA) biplot of hybrid entries used in the current study.

### 4.2 Identification of optimal phenotyping environment for traits affecting lodging

Several factors can cause lodging in crop plants; one such factor is the application of water to crop plants, which can reduce soil density and cause lodging by weakening soil strength (Piñera-Chavez et al., 2016). To identify the optimal environment, four lodging-susceptible and three lodging-tolerant hybrid entries were evaluated for plant culm strength and other agro-morphological traits under two conditions: well-watered (WW) and water-stressed (WS; no irrigation after grain set) during the summer season of 2017 (Experiment II). At anthesis, traits show no significant differences between lodging tolerant and lodging susceptible entries under well-watered condition (**Table 8**). The overall mean dry weight of the main stem per tiller shoot (DWMS/tiller shoot) of tolerant entries was significantly higher than that of susceptible entries at the milky-grain stage, indicating that lodging-tolerant entries had a higher dry weight of the main culm than lodging-susceptible entries under well-watered conditions during the summer season of 2017 (Experiment II) (**Table 9**). Again, under well-watered conditions, the overall mean PCS (*P* ≤ 0.05) and PG (*P* ≤ 0.01) values of lodging-tolerant entries were greater than those of lodging-susceptible entries at the grain-maturity stage (**Table 10**). No significant differences in traits were found between lodging-tolerant and lodging-susceptible entries under water-stressed conditions across the anthesis, milky-grain, and grain-maturity stages (**Tables 8**–**10**). A study by Nakhforoosh et al. (2020) reported that dry/water-stressed conditions could not differentiate between lodging susceptibility and lodging tolerance in oat multi-environment registration trials or provincial variety-testing trials. The mean culm wall width (CWW; *P* ≤ 0.05) and CRA (*P* ≤ 0.01) of susceptible entries under WW conditions were significantly greater than those under WS conditions at the anthesis stage (**Table 11**). Again, at the anthesis stage, the mean plant culm strength (PCS; *P* ≤ 0.05) and dry weight of the main stem (*P* ≤ 0.05) of susceptible and tolerant entries under WW conditions were significantly greater than those under WS conditions (**Table 11**, **Figure 4**). At the milky-grain stage, the mean PCS (*P* ≤ 0.05), DWMS (*P* ≤ 0.05), and CD (*P* ≤ 0.05) of lodging-susceptible entries under WW conditions were significantly greater than those under WS conditions. The mean internode length (INL) of tolerant entries under WW conditions was significantly greater (P ≤ 0.01) than that under WS conditions. The mean culm wall width (CWW) of lodging-tolerant entries showed significant differences (P ≤ 0.05) between WW and WS conditions, whereas the PH/culm weight of lodging-susceptible entries showed significant mean differences between WW and WS conditions (**Table 11**). At the grain-maturity stage, the mean plant height per culm weight (PH/culm weight) of both lodging-tolerant (*P* ≤ 0.01) and lodging-susceptible (*P* ≤ 0.05) entries showed significant differences between WW and WS conditions. Again, at the grain-maturity stage, the mean PCS (*P* ≤ 0.05 and *P* ≤ 0.01), DWMS (*P* ≤ 0.05), and dry weight of tillers (DW of tillers) (*P* ≤ 0.05) of lodging-tolerant and lodging-susceptible entries under WW conditions were significantly greater than those under WS conditions (**Table 11**, **Figure 4**).

**Table 8:** Lodging-tolerance-associated traits in pearl millet evaluated at the anthesis stage during the summer season of 2017 (Experiment II) at ICRISAT, Patancheru.

Well-watered											
Genotypic code	Lodging reactions	PCS (kPa)	INL (cm)	CD (cm)	CWW (mm)	CRA (°)	DW of MS (g)	DW of MS/tiller shoot	DW of tillers (g)	PH/INL weight	PH/culm weight
PMCHT05	Tolerant	192	11.06	10.25	0.61	57.83	21.04	5.16	4.65	16.34	10.42
PMCHT06	Tolerant	165.8	10.89	9.28	0.77	59.17	20.15	4.35	5.48	13.82	9.65
PMCHT07	Tolerant	201.87	11.19	9.43	0.67	57.59	21.62	5.29	4.89	14.63	9.83
Average		186.56	11.05	9.65	0.68	58.20	20.94	4.93	5.01	14.93	9.97
PMCHT11	Susceptible	175.73	9.54	9.35	0.6	60.33	18.09	2.5	7.89	17.77	11.46
PMCHT14	Susceptible	202.33	12.1	9.84	0.63	57.53	18.08	3.86	5.88	12.91	12.22
PMCHT15	Susceptible	201.07	12.08	10.17	0.62	54.2	23.68	4.07	5.04	14.92	9.18
PMCHT16	Susceptible	162.2	10.23	9.00	0.67	59.57	20.14	4.13	5.12	14.89	9.39
Average		185.33	10.99	9.59	0.63	57.91	20.00	3.64	5.98	15.12	10.56
t-test		0.08	0.09	0.16	1.09	0.20	0.68	2.67	1.38	0.15	0.75
p-value		0.94	0.93	0.88	0.39	0.86	0.55	0.06	0.26	0.88	0.51
Critical Difference at 5%		131.78	3.46	1.97	0.24	13.84	10.28	5.05	4.53	8.34	5.71
Coefficient of Variation (%)		19.9	8.8	5.9	10.9	6.9	14.9	29.8	23.2	16.9	16.2
Water-stressed											
PMCHT05	Tolerant	151.93	11.06	10.94	0.69	52.83	16.4	5.66	22.47	14	9.12
PMCHT06	Tolerant	116.27	10.75	8.72	0.57	51.5	11.54	1.72	11.54	13.73	15.12
PMCHT07	Tolerant	136.4	12.16	10.75	0.59	54.5	13.74	3.6	18.79	13.93	10.55
Average		134.87	11.32	10.14	0.62	52.94	13.89	3.66	17.60	13.89	11.60
PMCHT11	Susceptible	124.47	9.56	9.29	0.5	57.33	6.93	2.58	5.21	14.65	13.51
PMCHT14	Susceptible	140.33	11.98	10.29	0.56	53.33	9.98	2.67	9.98	11.28	11.85
PMCHT15	Susceptible	143.87	12.46	9.87	0.54	50.1	12.65	2.88	12.65	12.59	11.71
PMCHT16	Susceptible	144.47	10.85	9.58	0.63	56.5	8.26	3.43	10.1	14.8	11.24
Average		138.29	11.21	9.76	0.56	54.32	9.46	2.89	9.49	13.33	12.08
t-test		0.30	0.14	0.51	1.29	0.74	2.37	0.67	2.28	0.65	0.26
p-value		0.79	0.89	0.66	0.29	0.50	0.08	0.57	0.15	0.56	0.82
Critical Difference at 5%		94.78	16.26	2.46	5.92	2.39	0.17	3.70	8.13	7.16	4.87
Coefficient of Variation (%)		19.9	9.5	7.0	8.0	4.4	28.10	22.0	13.1	10.50	17.30

PCS, Plant culm strength; INL, Internode length; CD, Culm diameter; CWW, Culm wall width; CRA, Crown root angle; DWMS, Dry weight of main stem; DWMS/tiller shoot, Dry weight of main stem per tiller shoot; DW of tillers, Dry weight of tillers; PH/INL weight, Plant height per internode length weight; PH/culm weight, Plant height per culm weight.

**Table 9:** Lodging-tolerance-associated traits in pearl millet evaluated at the milky-grain stage during the summer season of 2017 (Experiment II) at ICRISAT, Patancheru.

Well-watered											
Genotypic code	Lodging reactions	PCS (kPa)	INL (cm)	CD (cm)	CWW (mm)	CRA (°)	DW of MS (g)	DW of MS/tiller shoot	DW of tillers (g)	PH/INL weight	PH/culm weight
PMCHT05	Tolerant	197.13	11.2	10.4	0.69	59.13	23.24	5.8	5.61	13.87	8.03
PMCHT06	Tolerant	189.6	11.12	10.29	0.62	60	22.97	5.48	9.22	13.63	8.27
PMCHT07	Tolerant	151.33	11.29	10.47	0.66	59.17	26.82	5.8	3.88	14.35	7.52
Average		179.35	11.20	10.39	0.66	59.43	24.34	5.69	6.24	13.95	7.94
PMCHT11	Susceptible	135.47	10.3	10.17	0.61	61.83	18.23	3.06	7.66	14.17	9.58
PMCHT14	Susceptible	128.47	11.72	10.17	0.63	62.9	17.95	3.81	4.9	12.25	8.62
PMCHT15	Susceptible	163.53	11.9	10.39	0.64	59.87	24.16	3.92	7.3	13.22	7.52
PMCHT16	Susceptible	208.4	10.44	10.64	0.68	56.8	24.84	2.99	8.09	16.38	6.87
Average		158.97	11.09	10.34	0.64	60.35	21.30	3.45	6.99	14.01	8.15
t-test		0.89	0.27	0.36	0.67	0.67	1.37	8.45**	0.43	0.06	0.33
p-value		0.43	0.81	0.74	0.55	0.55	0.24	0.001	0.71	0.96	0.77
Critical Difference at 5%		114.16	3.74	2.18	0.17	9.03	13.01	3.53	3.01	4.67	3.60
Coefficient of Variation (%)		20.5	9.5	6.1	8.5	4.3	16.7	21.2	14.5	10	12.5
Water-stressed											
PMCHT05	Tolerant	83.8	10.25	10.27	0.81	54.6	15.73	4.39	2.33	10.72	14.33
PMCHT06	Tolerant	107.67	9.9	9.58	0.78	59.2	20.27	7.27	3.02	9.46	15.51
PMCHT07	Tolerant	110.53	10.09	9.32	0.77	55.3	18.11	6.92	3.53	11.79	15.86
Average		100.67	10.08	9.72	0.79	56.37	18.04	6.19	2.96	10.66	15.23
PMCHT11	Susceptible	79.27	10.14	8.81	0.6	64.6	12.19	2.87	6.02	17.25	13.43
PMCHT14	Susceptible	107.13	10.99	9.86	0.67	58.47	15.19	5.86	4.49	14.96	12.61
PMCHT15	Susceptible	105.87	11.89	9.64	0.81	60.1	16.36	5.09	3.51	10.61	13.18
PMCHT16	Susceptible	111.53	9.85	9.82	0.71	57.7	17.08	5.25	3.34	10.42	16.04
Average		100.95	10.72	9.53	0.70	60.22	15.21	4.77	4.34	13.31	13.82
t-test		0.03	1.36	0.51	1.96	1.83	1.67	1.28	1.95	1.45	1.59
p-value		0.98	0.27	0.64	0.14	0.14	0.17	0.29	0.12	0.24	0.19
Critical Difference at 5%		97.21	9.62	2.21	0.35	11.11	9.62	4.91	2.21	5.30	11.01
Coefficient of Variation (%)		27.7	7.9	6.7	13.0	5.4	17.0	22.5	18.2	10.8	26.4

PCS, Plant culm strength; INL, Internode length; CD, Culm diameter; CWW, Culm wall width; CRA, Crown root angle; DWMS, Dry weight of main stem; DWMS/tiller shoot, Dry weight of main stem per tiller shoot; DW of tillers, Dry weight of tillers; PH/INL weight, Plant height per internode length weight; PH/culm weight, Plant height per culm weight. ***, *P* ≤ 0.001.

**Table 10:** Lodging-tolerance-associated traits in pearl millet evaluated at the grain-maturity stage during the summer season of 2017 (Experiment II) at ICRISAT, Patancheru.

Well-watered												
Genotypic code	Lodging reactions	PCS (kPa)	INL (cm)	CD (cm)	CWW (mm)	CRA (°)	DW of MS (g)	DW of MS/tiller shoot	DW of tillers (g)	PH/INL weight	PH/culm weight	PG (mm)
PMCHT05	Tolerant	175.33	10.46	9.69	0.7	56.03	21.17	6.76	5.05	14.48	7.87	27.41
PMCHT06	Tolerant	180.53	8.98	9.94	0.76	55.07	23.57	4.99	3.31	17.09	7.81	27.67
PMCHT07	Tolerant	206.8	9.96	11.01	0.66	56.8	27.43	6.21	6.69	17.54	7.24	27.77
Average		187.55	9.80	10.21	0.71	55.97	24.06	5.99	5.02	16.37	7.64	27.62
PMCHT11	Susceptible	153.67	8.97	10.04	0.64	61.3	19.24	3.04	6.59	16.2	8.95	25.91
PMCHT14	Susceptible	170.2	11.16	10.63	0.7	56.93	21.81	6.02	4.06	13	7.93	26.43
PMCHT15	Susceptible	165.13	11.58	9.94	0.64	58.13	23.74	7.48	4.32	13.68	7.85	25.2
PMCHT16	Susceptible	163.73	9.23	10.06	0.69	51.43	25.22	9.55	4.26	17.99	7.87	25.77
Average		163.18	10.24	10.17	0.67	56.95	22.50	6.52	4.81	15.22	8.15	25.83
t-test		2.67*	0.55	0.11	1.18	0.46	0.70	0.37	0.18	0.77	1.53	6.52**
p-value		0.04	0.61	0.93	0.32	0.67	0.54	0.74	0.87	0.48	0.20	0.007
Critical Difference at 5%		83.26	3.25	2.91	0.42	11.90	9.07	4.88	3.25	4.71	2.91	2.32
Coefficient of Variation (%)		14.3	9.0	8.3	18.0	5.9	11.4	20.6	19.0	9.2	3.2	8.3
Water-stressed												
PMCHT05	Tolerant	98.67	8.05	10.66	0.62	53.23	17.39	8.64	2.54	16.4	9.63	24.44
PMCHT06	Tolerant	110.07	9.38	9.5	0.7	60.19	14.8	15.59	1.22	16.62	10.82	26.36
PMCHT07	Tolerant	148.4	8.91	10.38	0.81	58.9	19.47	8.25	2.96	18.15	9.32	26.48
Average		119.05	8.78	10.18	0.71	57.44	17.22	10.83	2.24	17.06	9.92	25.76
PMCHT11	Susceptible	124.67	8.84	10.65	0.71	56.97	16.12	3.98	4.15	16.88	10.68	23.96
PMCHT14	Susceptible	105	9.95	9.8	0.67	50.62	14.65	9.86	2.01	13.63	10.15	22.93
PMCHT15	Susceptible	137.4	9.62	9.62	0.69	56.3	20.91	7.62	3.28	17.11	9.11	24.38
PMCHT16	Susceptible	110.73	8.4	8.89	0.82	54.43	17.34	9.37	1.91	19.53	10.4	23.96
Average		119.45	9.20	9.74	0.72	54.58	17.26	7.71	2.84	16.79	10.09	23.81
t-test		0.02	0.80	0.88	0.19	1.11	0.02	1.14	0.80	0.20	0.28	2.68
p-value		0.98	0.47	0.43	0.86	0.35	0.99	0.34	0.47	0.85	0.79	0.12
Critical Difference at 5%		89.80	7.16	3.88	2.42	2.84	0.21	3.51	13.12	3.36	3.91	6.78
Coefficient of Variation (%)		21.9	11.2	8.3	8.0	6.7	12.1	12.3	26.4	11.8	9.5	4.5

PCS, Plant culm strength; INL, Internode length; CD, Culm diameter; CWW, Culm wall width; CRA, Crown root angle; DWMS, Dry weight of main stem; DWMS/tiller shoot, Dry weight of main stem per tiller shoot; DW of tillers, Dry weight of tillers; PH/INL weight, Plant height per internode length weight; PH/culm weight, Plant height per culm weight; PG, Panicle girth. **, *P* ≤ 0.01; *, *P* ≤ 0.05.

**Table 11:** Comparison among lodging-tolerance-associated traits in pearl millet evaluated under well-watered and water-stressed conditions at anthesis, milky-grain, and grain-maturity stages during the summer season of 2017 (Experiment II) at ICRISAT, Patancheru.

Traits	Anthesis		Milky-grain		Grain-maturity	
	Susceptible	Tolerant	Susceptible	Tolerant	Susceptible	Tolerant
PCS (kPa)	0.02*	0.02*	0.03*	0.06	0.02*	0.006**
CRA (°)	0.00**	0.06	0.94	0.12	0.32	0.59
DWMS/tiller shoot	0.09	0.30	0.10	0.66	0.29	0.23
DWMS (g)	0.00**	0.03*	0.01*	0.08	0.03*	0.05*
DW of tillers (g)	0.21	0.07	0.08	0.19	0.009**	0.03*
INL (cm)	0.27	0.51	0.12	0.006**	0.07	0.34
PH/INL weight	0.07	0.26	0.47	0.09	0.10	0.43
PH/stem weight	0.10	0.50	0.02*	0.09	0.006**	0.03*
CD (cm)	0.48	0.47	0.03*	0.15	0.35	0.95
CWW (mm)	0.01*	0.50	0.24	0.01*	0.19	0.97

PCS, Plant culm strength; CRA, Crown root angle; DWMS/tiller shoot, Dry weight of main stem per tiller shoot; DWMS, Dry weight of main stem; DW of tillers, Dry weight of tillers; INL, Internode length; PH/INL weight, Plant height per internode length weight; PH/Stem weight, Plant height per stem weight; CD, Culm diameter; CWW, Culm wall width; PG, Panicle. **P ≤ 0.01; *P ≤ 0.05.

**Figure 4:**
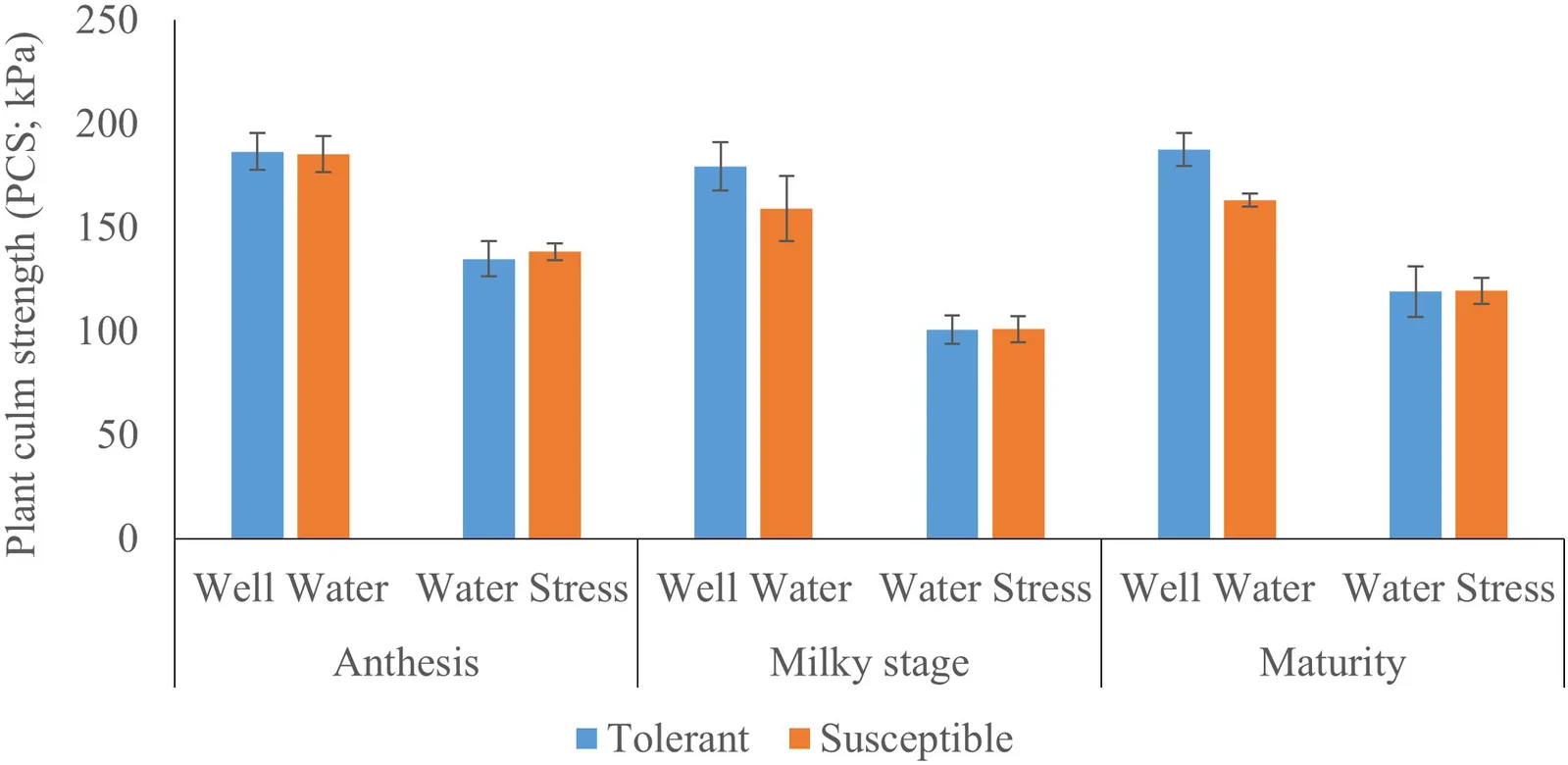
Plant culm strength values of tolerant and susceptible hybrid entries under well-watered and water-stressed conditions at the anthesis, milky-grain, and grain-maturity stages. Error bars indicate the standard error of the mean for hybrid entries.

Our study found that traits like culm wall width, culm diameter, internode length, plant culm strength, dry weight of the main stem, dry weight of tillers, and PH/culm weight of lodging-tolerant and lodging-susceptible entries showed significant differences between WW and WS conditions, indicating that the WW condition can provide an optimal environment for phenotyping traits linked to lodging tolerance in pearl millet.

### 4.3 Identification of plant growth stages associated with lodging

Lodging tolerance varies as plants develop because of a series of physiological changes that occur during plant growth stages. Numerous studies have suggested that crops are more susceptible to lodging, particularly during the milky-grain and grain-maturity stages (Tian et al., 2018; Parvej et al., 2020). Lodging in pearl millet began to increase during and/or after anthesis due to the transport of carbohydrates from the stalk to the panicle during grain formation, thereby reducing the mechanical strength of plants. The grain and/or panicle weight gradually increases during the milky-grain stage and raises the plant’s center of gravity. At the grain-maturity stage, the plant starts to lose moisture and carbohydrates, lowering the center of gravity, while panicle weight does not increase.

Our study found that most of the traits showed significant differences between susceptible and tolerant entries at milky-grain and maturity stages across years, indicating that lodging tolerance-linked traits can be phenotyped at the milky-grain and grain-maturity stages which affects grain yield and mechanical harvesting. A study conducted by Qun et al. (2020) on maize found that no specific lodging-resistance indicator was identified during the R1 (silking) stage, while ear height and high dry matter per unit length of the basal internodes at the R3 (milk) stage and center-of-gravity height and the mechanical strength of the basal internodes at the R6 (maturity) stage were found to be stage-specific lodging-resistance indicators. These traits can be measured at the corresponding growth stages to evaluate lodging resistance in maize. Earlier studies have also reported that lodging incidence at the reproductive stage resulted in greater yield reduction (Piñera-Chavez et al., 2016).

## 5 Conclusions

The two traits, plant culm strength (measured using a digital plant stem strength meter) and panicle girth, were found to differentiate well between lodging-tolerant and lodging-susceptible entries of pearl millet. Plant culm strength is a scorable, weather-independent, and non-destructive trait, making it more robust for screening breeding materials for lodging tolerance on a large scale under field conditions. In the present study, plant culm strength showed a high positive correlation with culm diameter at the grain-maturity stage in lodging-tolerant entries, indicating that a thicker culm plays an important role in the lodging-tolerance mechanism. Principal component analysis clearly differentiated lodging-tolerant and lodging-susceptible entries. This study identified the well-watered environment as optimal for phenotyping lodging-tolerance-linked traits compared with the WS condition. Assessment of lodging-tolerance-linked traits at different plant growth stages (anthesis, milky-grain, and grain-maturity) indicated that the milky-grain and grain-maturity stages were ideal for screening for lodging tolerance in pearl millet. These findings from preliminary studies can help researchers and breeders strengthen the phenotyping protocol for screening for lodging tolerance and identify lodging-tolerant breeding materials in pearl millet.

## Data Availability

The original contributions presented in the study are included in the article/**Supplementary Material**, further inquiries can be directed to the corresponding author/s.

## Supplementary material

Supplementary Table 1 Genotypic and company codes and lodging reactions of 29 pearl millet hybrids evaluated over 4 years (2016–2019).

Supplementary Table 2 Physical and chemical properties of Alfisol soils at ICRISAT, Hyderabad, Telangana.

Supplementary Table 3 Correlation between plant culm strength and agro-morphological and biochemical traits in Experiments I, II, IV, and V.

Supplementary Figure 1 Pearl millet lodging in the field.
