# Supplementary material for: Phenotyping for lodging tolerance in pearl millet: identification of optimal environment, plant stage, and potential traits
Source: Front Plant Sci. 2026 Sep 28;17:1879275. doi: 10.3389/fpls.2026.1879275 (PMC13655672; doi:10.3389/fpls.2026.1879275)
Supplement: Supplementary file 1 [file DataSheet2.pdf]

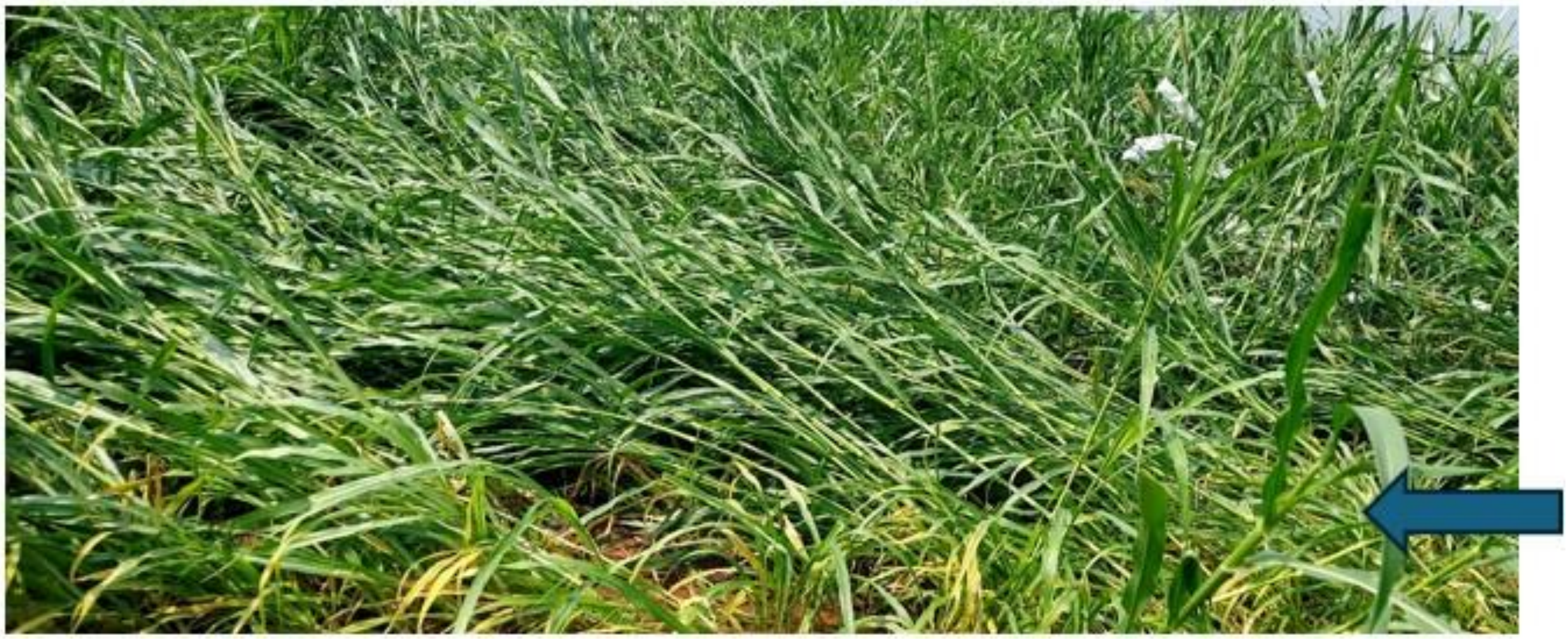

Supplementary Figure 1. Pearl millet lodging in the field.
