# Supplementary material for: Phenotyping for lodging tolerance in pearl millet: identification of optimal environment, plant stage, and potential traits
Source: Front Plant Sci. 2026 Sep 28;17:1879275. doi: 10.3389/fpls.2026.1879275 (PMC13655672; doi:10.3389/fpls.2026.1879275)
Supplement: Supplementary file 2 [file DataSheet1.docx]

**Supplementary Table 1.** Genotypic and company codes, lodging reactions of 29 pearl millet hybrids evaluated over 4 years (2016-2019).

| **S.No.** | **Genotype Code** | **Company Code** | **Lodging reactions** | **Evaluated seasons/years** | | | | | |
| --- | --- | --- | --- | --- | --- | --- | --- | --- | --- |
|  |  |  |  | **Rainy 2016 (Well water)** | **Summer 2017** | | **Rainy 2018 (Well water)** | **Summer 2019 (Well water)** | **Rainy 2019 (Well water)** |
|  |  |  |  |  | **Well water** | **Water stress** |  |  |  |
| 1 | ^†^PMCHT01 | C-11 | Tolerant | √ |  |  |  | √ |  |
| 2 | PMCHT02 | C-12 | Tolerant | √ |  |  | √ | √ |  |
| 3 | PMCHT03 | C-21 | Tolerant | √ |  |  | √ |  |  |
| 4 | PMCHT04 | C-25 | Tolerant | √ |  |  | √ |  |  |
| 5 | PMCHT05 | C-31 | Tolerant |  | √ | √ | √ | √ | √ |
| 6 | PMCHT06 | C-32 | Tolerant |  | √ | √ | √ |  | √ |
| 7 | PMCHT07 | C-33 | Tolerant |  | √ | √ | √ | √ | √ |
| 8 | PMCHT08 | C-41 | Tolerant |  |  |  | √ | √ | √ |
| 9 | PMCHT09 | C-42 | Tolerant |  |  |  | √ |  |  |
| 10 | PMCHT10 | C-51 | Tolerant |  |  |  | √ | √ | √ |
| 11 | PMCHT11 | C-22 | Susceptible | √ | √ | √ | √ | √ | √ |
| 12 | PMCHT12 | C-23 | Susceptible | √ |  |  | √ |  |  |
| 13 | PMCHT13 | C-24 | Susceptible | √ |  |  |  |  |  |
| 14 | PMCHT14 | C-34 | Susceptible |  | √ | √ |  |  |  |
| 15 | PMCHT15 | C-35 | Susceptible |  | √ | √ | √ |  |  |
| 16 | PMCHT16 | C-36 | Susceptible |  | √ | √ | √ | √ |  |
| 17 | PMCHT17 | C-37 | Expected to be tolerant |  |  |  |  |  | √ |
| 18 | PMCHT18 | C-52 | Expected to be tolerant |  |  |  |  |  | √ |
| 19 | PMCHT19 | C-61 | Expected to be tolerant |  |  |  |  |  | √ |
| 20 | PMCHT20 | C-62 | Expected to be tolerant |  |  |  |  |  | √ |
| 21 | PMCHT21 | C-63 | Expected to be tolerant |  |  |  |  |  | √ |
| 22 | PMCHT22 | C-64 | Expected to be tolerant |  |  |  |  |  | √ |
| 23 | PMCHT23 | C-71 | Expected to be tolerant |  |  |  |  |  | √ |
| 24 | PMCHT24 | C-72 | Expected to be tolerant |  |  |  |  |  | √ |
| 25 | PMCHT25 | C-81 | Expected to be tolerant |  |  |  |  |  | √ |
| 26 | PMCHT26 | C-82 | Expected to be tolerant |  |  |  |  |  | √ |
| 27 | PMCHT27 | C-91 | Expected to be tolerant |  |  |  |  |  | √ |
| 28 | PMCHT28 | C-10 | Expected to be tolerant |  |  |  |  |  | √ |
| 29 | PMCHT29 | C-111 | Expected to be tolerant |  |  |  |  |  | √ |

^†^PMCHT: Pearl millet collaborative hybrids trial; √: indicates that the entry was involved in the experiment

**Supplementary Table 2.** Physical and chemical properties of Alfisol soils at ICRISAT, Hyderabad, Telangana.

| Horizon | Depth (cm) | Particle size distribution (% of <2 mm) | | | | Organic carbon (%) | CaCO_3 (%)_ | BD^a^ Mg m^-3^ | sHC^b^ cm hr^-1^ | Water retension (%) | | pH | EC^c^ | Extractable bases | | | | CEC^d^ | | Base saturation (%) | ESP^e^ |
| --- | --- | --- | --- | --- | --- | --- | --- | --- | --- | --- | --- | --- | --- | --- | --- | --- | --- | --- | --- | --- | --- |
|  |  | Sand (0.02-2 mm) | Silt (0.02-0.002 mm) | Clay (<0.002 mm) | Fine clay (<0.0002) |  |  |  |  | 33 kPa | 1500 kPa | 1: 2.5 water | (1: 2.5 water) dSm^-1^ | Ca Mg Na K | | | | Soil | Clay |  |  |
|  |  |  |  |  |  |  |  |  |  |  |  |  |  | cmol(+) kg^-1^ | | | |  |  |  |  |
| A1 | 0–4 | 66.7 | 15.4 | 17.9 | 12.5 | 3.1 | 0.6 | 1.35 | 3.63 | 24 | 15 | 6.4 | 0.17 | 4.2 | 3.8 | 0.8 | 1.2 | 10 | 36 | 12 | 8 |
| A2 | 4–11 | 74.7 | 10.8 | 14.5 | 11.0 | 1.6 | 0.4 | 1.35 | 3.91 | 24 | 14 | 6.2 | 0.05 | 3.3 | 3.4 | 0.6 | 0.3 | 10 | 24 | 76 | 6 |
| Bt1 | 11–38 | 50.6 | 5.3 | 44.1 | 37.3 | 1.0 | 0.6 | 1.60 | 3.92 | 31 | 23 | 6.1 | 0.03 | 8.3 | 3.3 | 0.7 | 0.3 | 11 | 26 | 111 | 6 |
| Bt2 | 38–65 | 35.9 | 11.5 | 52.6 | 41.9 | 0.7 | 0.8 | 1.71 | 3.82 | 35 | 28 | 6.1 | 0.05 | 12.5 | 5.2 | 0.8 | 0.3 | 18 | 35 | 103 | 4 |
| Bt3 | 65–79 | 34.8 | 12.0 | 53.2 | 40.9 | 0.6 | 0.8 | 1.70 | 3.91 | 39 | 30 | 6.0 | 0.06 | 12.4 | 5.9 | 0.7 | 0.4 | 26 | 40 | 92 | 3 |
| BC | 79–109 | 50.4 | 14.4 | 35.2 | 25.9 | 0.4 | 0.9 | 1.72 | 3.62 | 28 | 21 | 6.4 | 0.05 | 14.8 | 5.7 | 0.8 | 0.2 | 23 | 66 | 93 | 4 |
| C | 109–163 | 61.1 | 16.4 | 22.5 | 17.5 | 0.2 | 0.9 | 1.84 | 3.57 | 21 | 15 | 6.6 | 0.04 | 16.3 | 5.0 | 0.9 | 0.3 | 22 | 100 | 101 | 4 |

^a^BD, bulk density; ^b^sHC, saturated hydraulic conductivity; ^c^EC, electrical conductivity; ^d^CEC, cation exchange capacity; ^e^ESP, exchangeable sodium percentage.

Note: Soil characteristics data adapted from Bhattacharyya et al. (2016).

Supplementary Table 3. Correlation between plant culm strength and agro-morphological and biochemical traits of Experiments-I, II, IV, and V. (contd..,,)

| **Experiment-I** | **Crop Stage** | **Condition** | **Reaction to lodging** | **PH (cm)** | **NT** | **INL (1-2)** | **INL (2-3)** | **CD 1-2** | **CD (2-3)** | **CWW (mm)** | **PL (cm)** | **PG (mm)** | **CRA (˚)** | **DWMS/tiller shoots** | **PH/stem weight** | **RPS (cm)** | **FSB (g)** | **DSB (g)** | **FRB (g)** | **DRB (g)** | **GY (g/plant)** | **DW of MS (g)** | **DW of tillers (g)** |
| --- | --- | --- | --- | --- | --- | --- | --- | --- | --- | --- | --- | --- | --- | --- | --- | --- | --- | --- | --- | --- | --- | --- | --- |
|  | Maturity | WW | Tolerant | 0.9 | NA | NA | NA | NA | NA | NA | 0.23 | -0.73 | NA | -0.51 | -0.39 | NA | NA | NA | NA | NA | NA | NA | NA |
|  | Maturity | WW | Susceptible | -0.91 | NA | NA | NA | NA | NA | NA | -0.37 | -0.46 | NA | -0.15 | 0.23 | NA | NA | NA | NA | NA | NA | NA | NA |
| **Experiment-II** | Maturity | WW | Tolerant | NA | NA | 0.03 | NA | **1.00**** | NA | -0.7 | NA | 0.81 | 0.74 | 0.06 | -1 | NA | NA | NA | NA | NA | NA | 0.97 | 0.77 |
|  | Maturity | WW | Susceptible | NA | NA | 0.75 | NA | 0.61 | NA | 0.67 | NA | 0.21 | -0.49 | 0.58 | -0.89 | NA | NA | NA | NA | NA | NA | 0.56 | -0.95 |
|  | Maturity | WS | Tolerant | NA | NA | 0.38 | NA | 0.07 | NA | 0.98 | NA | 0.71 | 0.54 | -0.34 | -0.48 | NA | NA | NA | NA | NA | NA | 0.69 | 0.51 |
|  | Maturity | WS | Susceptible | NA | NA | 0.06 | NA | 0.29 | NA | -0.25 | NA | 0.83 | 0.82 | -0.57 | -0.61 | NA | NA | NA | NA | NA | NA | 0.83 | 0.74 |
|  | Milky | WW | Tolerant | NA | NA | -0.8 | NA | -0.69 | NA | 0.07 | NA | NA | 0.32 | -0.36 | 0.89 | NA | NA | NA | NA | NA | NA | -0.98 | 0.64 |
|  | Milky | WW | Susceptible | NA | NA | -0.32 | NA | **1.00**** | NA | 0.93 | NA | NA | **-1.00**** | -0.42 | -0.88 | NA | NA | NA | NA | NA | NA | 0.9 | 0.67 |
|  | Milky | WS | Tolerant | NA | NA | -0.78 | NA | -0.99 | NA | -0.99 | NA | NA | 0.54 | 0.98 | 0.99 | NA | NA | NA | NA | NA | NA | 0.83 | 0.94 |
|  | Milky | WS | Susceptible | NA | NA | 0.27 | NA | **0.98*** | **NA** | 0.68 | NA | NA | **-0.98*** | 0.95 | 0.32 | NA | NA | NA | NA | NA | NA | **0.96*** | -0.93 |
|  | Anthesis | WW | Tolerant | NA | NA | 0.98 | NA | 0.39 | NA | -0.8 | NA | NA | -0.99 | 0.99 | 0.46 | NA | NA | NA | NA | NA | NA | 0.99 | -0.85 |
|  | Anthesis | WW | Susceptible | NA | NA | 0.88 | NA | **0.96*** | NA | -0.46 | NA | NA | -0.78 | 0.22 | 0.28 | NA | NA | NA | NA | NA | NA | 0.26 | -0.19 |
|  | Anthesis | WS | Tolerant | NA | NA | 0.28 | NA | 0.93 | NA | 0.9 | NA | NA | 0.51 | 0.99 | -0.98 | NA | NA | NA | NA | NA | NA | 0.99 | 0.99 |
|  | Anthesis | WS | Susceptible | NA | NA | 0.8 | NA | 0.59 | NA | 0.75 | NA | NA | -0.58 | 0.66 | **-0.99*** | NA | NA | NA | NA | NA | NA | 0.67 | 0.93 |
| **Experiment-IV** | Maturity | WW | Tolerant | 0.29 | -0.09 | 0.24 | 0.01 | 0.28 | -0.12 | NA | 0.36 | 0.06 | 0.69 | NA | NA | 0.19 | 0.72 | 0.66 | 0.57 | -0.24 | 0.7 | NA | NA |
| **Experiment-V** | Maturity | WW | Tolerant & Expected to be tolerant | 0.45 | **-0.47*** | 0.25 | 0.19 | 0.33 | 0.43 | NA | 0.19 | -0.06 | 0.22 | NA | NA | -0.23 | 0.67** | NA | 0.21 | NA | NA | NA | NA |

**Note:** PH: Plant height; NT: Number of tillers; INL: Internode length; CD: Culm diameter; CWW: Culm wall width; PL: Panicle length; PG: Panicle girth; CRA: Crown root angle; DWMS/tiller shoots: Dry weight of main stem/tiller shoots; PH/stem weight: Plant height per stem weight; RPS: Root plate spread; FSB: Fresh shoot biomass; DSB: Dry shoot biomass; FRB: Fresh root biomass; DRB: Dry root biomass; GY: Grain yield; DW of MS: Dry weight of main stem; NA: Not available. **, *P* ≤ 0.01; *, *P* ≤ 0.05.

Supplementary Table 3. Correlation between plant culm strength and agro-morphological and biochemical traits of Experiments-I, II, IV, and V.

| **Experiment-I** | **Crop Stage** | **Condition** | **Reaction to lodging** | **PH/INL weight** | **Lodging (%)** | **LS** | **DW of leaf of MS** | **DW of stem of MS** | **DW of panicle of MS** | **DW of leaf tillers** | **DW of stem of tillers** | **DW of leaf PT** | **DW of stem of PT** | **DW of panicle of PT** | **TDW of MS** | **TDW of tillers** | **TDW of PT** | **ADL of MS (%)** | **HC of MS (%)** | **Cellulose of MS (%)** | **ADL of PT (%)** | **HC of PT (%)** | **Cellulose of PT (%)** |
| --- | --- | --- | --- | --- | --- | --- | --- | --- | --- | --- | --- | --- | --- | --- | --- | --- | --- | --- | --- | --- | --- | --- | --- |
|  | Maturity | WW | Tolerant | NA | -0.34 | NA | 0.74 | 0.68 | 0.5 | 0.68 | 0.15 | 0.27 | 0.69 | 0.81 | 0.62 | 0.59 | 0.74 | -0.35 | -0.46 | -0.92 | NA | NA | NA |
|  | Maturity | WW | Susceptible | NA | **1**** | NA | -0.63 | -0.52 | -0.27 | 0.6 | 0.98 | 0.18 | 0.5 | 0.76 | -0.43 | -0.23 | 0.69 | 0.25 | 0.14 | **1**** | 0.41 | 0.09 | 0.33 |
| **Experiment-II** | Maturity | WW | Tolerant | 0.73 | NA | NA | NA | NA | NA | NA | NA | NA | NA | NA | NA | NA | NA | NA | NA | NA | NA | NA | NA |
|  | Maturity | WW | Susceptible | -0.55 | NA | NA | NA | NA | NA | NA | NA | NA | NA | NA | NA | NA | NA | NA | NA | NA | NA | NA | NA |
|  | Maturity | WS | Tolerant | 0.99 | NA | NA | NA | NA | NA | NA | NA | NA | NA | NA | NA | NA | NA | NA | NA | NA | NA | NA | NA |
|  | Maturity | WS | Susceptible | 0.26 | NA | NA | NA | NA | NA | NA | NA | NA | NA | NA | NA | NA | NA | NA | NA | NA | NA | NA | NA |
|  | Milky | WW | Tolerant | -0.88 | NA | NA | NA | NA | NA | NA | NA | NA | NA | NA | NA | NA | NA | NA | NA | NA | NA | NA | NA |
|  | Milky | WW | Susceptible | 0.85 | NA | NA | NA | NA | NA | NA | NA | NA | NA | NA | NA | NA | NA | NA | NA | NA | NA | NA | NA |
|  | Milky | WS | Tolerant | 0.05 | NA | NA | NA | NA | NA | NA | NA | NA | NA | NA | NA | NA | NA | NA | NA | NA | NA | NA | NA |
|  | Milky | WS | Susceptible | -0.81 | NA | NA | NA | NA | NA | NA | NA | NA | NA | NA | NA | NA | NA | NA | NA | NA | NA | NA | NA |
|  | Anthesis | WW | Tolerant | 0.54 | NA | NA | NA | NA | NA | NA | NA | NA | NA | NA | NA | NA | NA | NA | NA | NA | NA | NA | NA |
|  | Anthesis | WW | Susceptible | -0.51 | NA | NA | NA | NA | NA | NA | NA | NA | NA | NA | NA | NA | NA | NA | NA | NA | NA | NA | NA |
|  | Anthesis | WS | Tolerant | 0.98 | NA | NA | NA | NA | NA | NA | NA | NA | NA | NA | NA | NA | NA | NA | NA | NA | NA | NA | NA |
|  | Anthesis | WS | Susceptible | -0.37 | NA | NA | NA | NA | NA | NA | NA | NA | NA | NA | NA | NA | NA | NA | NA | NA | NA | NA | NA |
| **Experiment-IV** | Maturity | WW | Tolerant | NA | NA | 0.02 | NA | NA | NA | NA | NA | NA | NA | NA | NA | NA | NA | NA | NA | NA | NA | NA | NA |
| **Experiment-V** | Maturity | WW | Tolerant & Expected to be tolerant | NA | NA | **-0.48*** | NA | NA | NA | NA | NA | NA | NA | NA | NA | NA | NA | NA | NA | NA | NA | NA | NA |

**Note:** PH/INL weight: plant height per internode length weight; LS: Lodging score; DW of leaf of MS: Dry weight of leaf of main stem; DW of stem of MS: Dry weight of stem of main stem; DW of panicle of MS: Dry weight of panicle of main stem; DW of leaf of tillers: Dry weight of leaf of tillers; DW of stem of tillers: Dry weight of stem of tillers; DW of stem of PT: Dry weight of stem of productive tiller; DW of panicle of PT: Dry weight of panicle of productive tiller; TDW of MS: Total dry weight of main stem; TDW of tillers: Total dry weight of tillers; TDW of PT: Total dry weight of productive tiller; DWMS/tiller shoots: Dry weight of main stem/tiller shoots; PH/stem weight: Plant height per stem weight; ADL of MS: Acid detergent lignin of main stem; HC of MS: Hemicellulose of main stem; Cellulose of MS: Cellulose of main stem; ADL of PT: Acid detergent lignin of productive tillers; HC of PT: Hemicellulose of productive tiller; Cellulose of PT: Cellulose of productive tiller; NA: Not available. **, *P* ≤ 0.01; *, *P* ≤ 0.05.
